# Neural dynamics of grip and goal integration during the processing of others’ actions with objects: An ERP study

**DOI:** 10.1038/s41598-020-61963-7

**Published:** 2020-03-19

**Authors:** Jérémy Decroix, Clémence Roger, Solène Kalénine

**Affiliations:** Univ. Lille, CNRS, UMR 9193 - SCALab - Sciences Cognitives et Sciences Affectives, F-59000 Lille, France

**Keywords:** Cognitive neuroscience, Social neuroscience, Psychology

## Abstract

Recent behavioural evidence suggests that when processing others’ actions, motor acts and goal-related information both contribute to action recognition. Yet the neuronal mechanisms underlying the dynamic integration of the two action dimensions remain unclear. This study aims to elucidate the ERP components underlying the processing and integration of grip and goal-related information. The electrophysiological activity of 28 adults was recorded during the processing of object-directed action photographs (e.g., writing with pencil) containing either grip violations (e.g. upright pencil grasped with atypical-grip), goal violations (e.g., upside-down pencil grasped with typical-grip), both grip and goal violations (e.g., upside-down pencil grasped with atypical-grip), or no violations. Participants judged whether actions were overall typical or not according to object typical use. Brain activity was sensitive to the congruency between grip and goal information on the N400, reflecting the semantic integration between the two dimensions. On earlier components, brain activity was affected by grip and goal typicality independently. Critically, goal typicality but not grip typicality affected brain activity on the N300, supporting an earlier role of goal-related representations in action recognition. Findings provide new insights on the neural temporal dynamics of the integration of motor acts and goal-related information during the processing of others’ actions.

## Introduction

Understanding the actions performed by others is a core ability of human beings^1,2^. Yet, actions are no mere movements but organised and goal-directed movements^3,4^. Thus, understanding others’ actions does not only imply the processing of the motor act (e.g., both dynamic and static components of reaching and grasping a bottle) but also the recognition of the actor’s goal (e.g., to drink or to move it away). Accordingly, observers mainly perceive others’ actions in terms of goals^5–8^ and may use different sources of information to this end (e.g., dynamic and static component of the motor act^9^; functional knowledge about objects^10^; contextual information^11,12^, among others). Although numerous studies have demonstrated that both information about the motor act and information about the goal of the actor are processed during the decoding of others’ actions, the precise role of the two types of information in action recognition remains debated.

Important theoretical accounts have highlighted the need to consider the processing of others’ actions as a dynamic phenomenon^3,13–15^ that cannot be fully uncovered without considering the temporal dynamics of motor acts and action goal decoding. The observation of several successive periods of stability in the brain activity (i.e. “micro-state”) when visually processing others’ actions^16,17^ further supports the idea that the decoding of others’ actions is a multistep process. In a recent behavioural experiment, we also demonstrated that the involvement of action goal and static motor act information in action recognition could be temporally dissociated^18^. Briefly presented action primes (66-ms) sharing the same goal as target actions facilitated the visual recognition of the target actions whereas 66 ms action primes sharing the same grip configuration did not. These data suggest that information related to action goals is processed first^19,20^. These results fit well with the recent predictive approaches of action recognition in which initial predictions about the action goal are thought to drive the recognition of the grip configuration^21–23^. Although previous behavioural results suggest that goal-related information may be processed earlier than motor act information when observers decode visual actions, a complete picture of the timing of goal and motor acts decoding is lacking. Moreover, the neural correlates of the dynamic activation of goal and motor act information during action recognition remain to be identified.

Electroencephalography (EEG) is well suited to investigate the neural temporal dynamics of action processing. In particular, the technique of Event Related Potential (ERP) has been used to deepen our understanding of the processes underlying the decoding of others’ actions. Several late ERP components have been related to the processing of visual actions. Modulations of the N400 component by the congruency between an action and the context in which it takes place (e.g., squeezing a lemon in the bathroom instead of the kitchen) have been repeatedly reported^24–30^. Such modulations have been interpreted as a marker of the integration between different pieces of action information^30^. Yet these studies usually involve semantic violations that encompass several pieces of action-relevant information at the same time (e.g., the motor act, the object, the visual context and the action goal). Then, it is difficult to identify the contribution of each piece of information to the modulations observed on the N400 component. More specifically related to the dissociation between action goals and motor acts, Bach, Gunter, Knoblich, Prinz and Friederici^31^ demonstrated similar N400 modulations when processing violations of action static motor act components (e.g., inserting a screwdriver in a screw with a matching versus mismatching orientation) and violations of action goals (e.g., inserting a screwdriver in a keyhole versus a screw, both with a matching orientation). Therefore, both motor acts and action goal dimensions may independently contribute to modulations of the N400 component. This further suggests that the two dimensions can be dissociated and reflected in the components of visual ERPs, hence complementing previous behavioural results^18,32^. Focusing on the recognition of hand-object actions (i.e., does the action picture display a typical use of the object), Chang *et al*.^33^ reported modulations of the N300 component by the processing of action violations (e.g., using a precision grip on an upright pencil versus using a power grasp on an upside-down pencil). Yet it is unclear whether N300 modulations reflect the effect of grip-congruency (related to the static motor act component), or the impossibility to use the object for its typical function (related to the action goal). In another EEG study^34^, the independent manipulation of the motor act component and the action goal revealed an earlier modulation of the P300 component for object-goal violations (e.g. using a nail on a hammer) in comparison to object-grip violations (e.g., grasping the hammer by its head instead of its handle). Importantly, the expected integration of goal information and motor act components (reflected by the statistical interaction between goal and grip violations) could not be observed on this ERP component. It should be noted, however, that participants were explicitly asked to judge whether either the goal or the grip of the action was correct. The authors themselves suggested that the absence of integration between object-grip and goal-related information might have been explained by the specific task demands. Thus, from these results, P300 modulations may not be related to the spontaneous recognition of observed actions, which should require at some point the integration between the two action dimensions. Together, previous ERP findings indicate that P300, N300 and N400 components reflect the processing of different dimensions of observed actions but their selective sensitivity to motor act components, goal-related information or the integration between the two dimensions remain to be identified.

Finally, the cognitive mechanisms underlying the modulations of the aforementioned ERPs unlikely reflect the initial stages of action processing. Indeed, they have been related to the access to manipulation knowledge (i.e., related to the grip configuration) and functional knowledge (i.e., related to the action goal) relevant to the use of the object (e.g., the N300 component^33^) or associated to the integration of the two action dimensions (e.g., the N400 component^30^). The processing of others’ actions clearly begins much earlier. Different EEG techniques have, for example, found that discriminating between grasp-to-move and grasp-to-use actions modulated brain activity as early as 60 ms of action processing^16,17,35^. Whether such early modulations can be related to actual action processing or merely reflect perceptual differences in the study design remains debated^13,36^. Moreover, early differences between visual actions performed with distinct goals have been, again, interpreted as evidence of early brain sensitivity to either motor act components^17^ or action goals^35^.

Therefore, the present study aimed at characterising the ERP correlates of action goal and motor act decoding at both early and late stages of action processing. The present paradigm used photographs of object-directed actions (e.g., writing with pencil) displaying a hand and a tool-object. Actions could be typical or not according to the typical use of the object by the introduction of grip violations (e.g. upright pencil grasped with power grip), goal violations (e.g., upside-down pencil grasped with precision grip), or, both grip and goal violations (e.g., upside-down pencil grasped with power grip). Grip violations did not prevent the performance of the typical goal of the action and vice versa, so that the two dimensions varied independently from one another. Importantly, object identity was kept constant across conditions and object-related knowledge was equally diagnostic of grip and goal typicality. The concept of pencil is both associated to the typical functional goal of writing and to the typical precision grip for using the object. Consequently, any differences in processing actions with grip and goal violations could not merely reflect differences in the activation of object knowledge between conditions but would rather relate to the activation of different action representations. In order to assess the spontaneous recognition of others’ actions, participants were not explicitly asked to pay attention to one or the other dimension. They were asked to evaluate, on each trial, whether the overall action was correct or not while EEG was recorded and had to provide a behavioural response when prompted (12% of trials). Analyses focused on ERPs time-locked to action photograph onset. Differences in ERP amplitude as a function of grip typicality (grip typical versus grip atypical, independently of goal typicality) and goal typicality (goal typical versus goal atypical, independently of grip typicality) were assumed to reflect the decoding of the grip and the decoding of the goal, respectively. Differences in ERP amplitude as a function of grip and goal congruence (grip and goal dimensions congruent versus incongruent, regardless of which dimension is correct and which is incorrect) were assumed to reflect the integration of grip and goal dimensions. We expected the integration between goal and grip dimensions to be visible on late ERP components (e.g., N300, N400), as the processing of incongruencies between different action dimensions has been especially detected at such timing^24,31,33,37^. In addition, we wanted to evaluate whether differences in the action photographs in terms of grip or goal visual information would be detected on earlier ERP components.

Overall, the identification of object-directed actions induced P100, N170, P300, N300 and N400 components. Mean peak amplitudes and peak latencies of the ERPs were analysed as a function of the typicality of grip and goal-related information and the congruence between the two dimensions. In brief, we found that the N400 component was sensitive to the congruence between grip and goal dimensions, reflecting the semantic integration of grip and goal dimensions at that stage of action processing. The separate processing of the two dimensions was visible from the earliest stages of action processing on the P100 and N170 components. Interestingly, goal typicality but not grip typicality affected the amplitude of the anterior N300 component before grip and goal integration on the N400, highlighting earlier post-perceptual processing of the goal compared to the grip dimension during action recognition.

## Results

Although participants were instructed to judge the action photograph on each trial, an explicit behavioural response was required on 12% of the trials only. This choice was made to avoid contamination of the EEG signal by the preparation of the motor response^38–40^. Overall mean accuracy on the 12% trials associated with a response prompt was 73%. The rather low performances in the task most likely reflect the important working memory load induced by the response procedure. Indeed, our previous experiments with similar tasks and design revealed high-level of accuracy in performing perceptual judgements^18,41^. Similarly, perceptual judgements were rather high (90%) in the pre-test (cf. Stimuli section below). Thus, accuracy in the present experiment unlikely reflect the perceptual judgement of the photographs. Therefore, errors were solely analysed to verify the absence of systematic bias in the task. Mean accuracies by condition were distributed as followed: Goal-typical Grip-typical *M* 72% +/− 45% *SD*, Goal-atypical Grip-typical *M* 75% +/− 43% *SD*, Goal-typical Grip-atypical *M* 66% +/− 47% *SD*, Goal-atypical Grip-atypical *M* 79% +/− 40% *SD*. A chi-square test for independence indicated that errors were equally distributed between conditions, χ^2^_3_ = 1.03, *p* = 0.79. Overall, we can be confident that participants performed correctly the task.

Mean peak amplitudes/peak latencies of the ERP components were analysed as a function of grip-typicality (Grip-typical versus Grip-atypical) and goal-typicality (Goal-typical versus Goal-atypical). Grip activation was statistically tested through the main effect of grip-typicality. Goal activation was statistically tested through the main effect of goal-typicality. The integration of the two dimensions (i.e. the sensitivity to the congruence between the two dimensions) was statistically tested through the interaction between grip-typicality and goal-typicality factors.

### Posterior P100, N170 and P300

**Figure 1:**
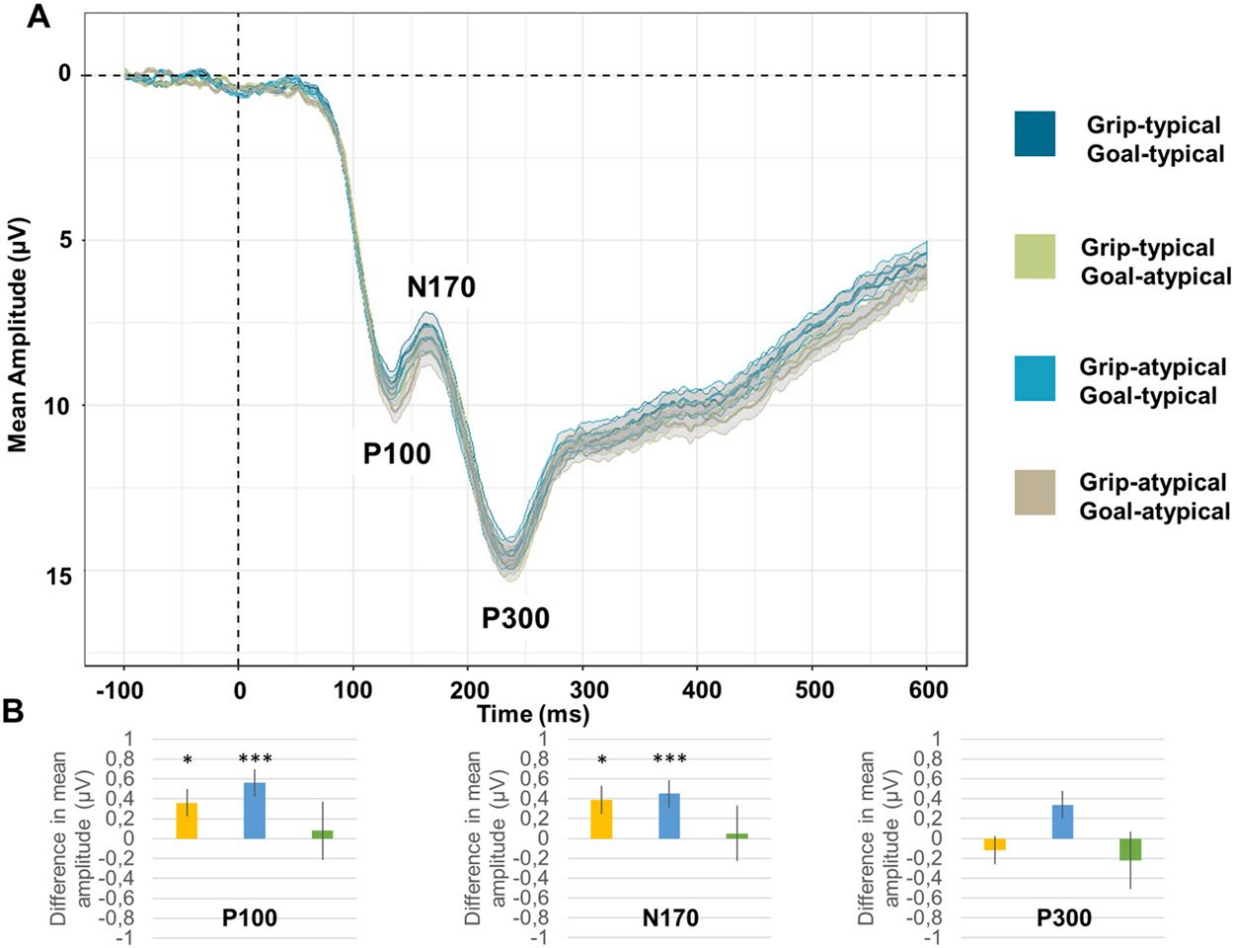
(**A**) ERP as a function of Grip typicality and Goal-typicality at the posterior site. Ribbons represent standard errors. (**B**) Mean estimates of the main effect of grip-typicality (yellow bar), main effect of goal-typicality (blue bar) and Grip x Goal interaction (green bar), for the P100, N170 and P300 components. Error bars represent standard errors. *p < 0.05; ***p < 0.001.

Analysis of mean peak amplitude of the P100 component revealed significant main effects of both Grip-typicality, *F*_1,81_ = 6.11, *p*_*corrected*_ = 0.046, *Westfall’s d* = 0.09, and Goal-typicality *F*_1,81_ = 15.27, *p*_*corrected*_ < 0.001, *Westfall’s d* = 0.15. In both cases, the P100 was more positive for the typical dimension than for the atypical dimension (Grip-atypical – Grip-typical = 0.36 µV, *SE* = 0.14; Goal-atypical – Goal-typical = 0.56 µV, *SE* = 0.14). On the N170 component, main effects were significant for both Grip-typicality *F*_1,78.04_ = 7.75, *p*_*corrected*_ = 0.020, *Westfall’s d* = 0.08, and Goal-typicality *F*_1,79.01_ = 10.32, *p*_*corrected*_ = 0.006, *Westfall’s d* = 0.09. In both cases, the N170 was more negative for the typical dimension than for the atypical dimension (Grip-atypical – Grip-typical = −0.39 µV, *SE* = 0.14; Goal-atypical – Goal-typical = −0.45 µV, *SE* = 0.14). The significance of the main effect of Goal-typicality on the P300 component did not survive the Bonferroni correction (*p*_*corrected*_ = 0.077). Results are displayed on Fig. 1.

The analysis of the peak latencies did not reveal any significant effects for the P100, N170 or P300 components (all *ps*_*uncorrected*_ > 0.145).

### Anterior N300 and N400

**Figure 2:**
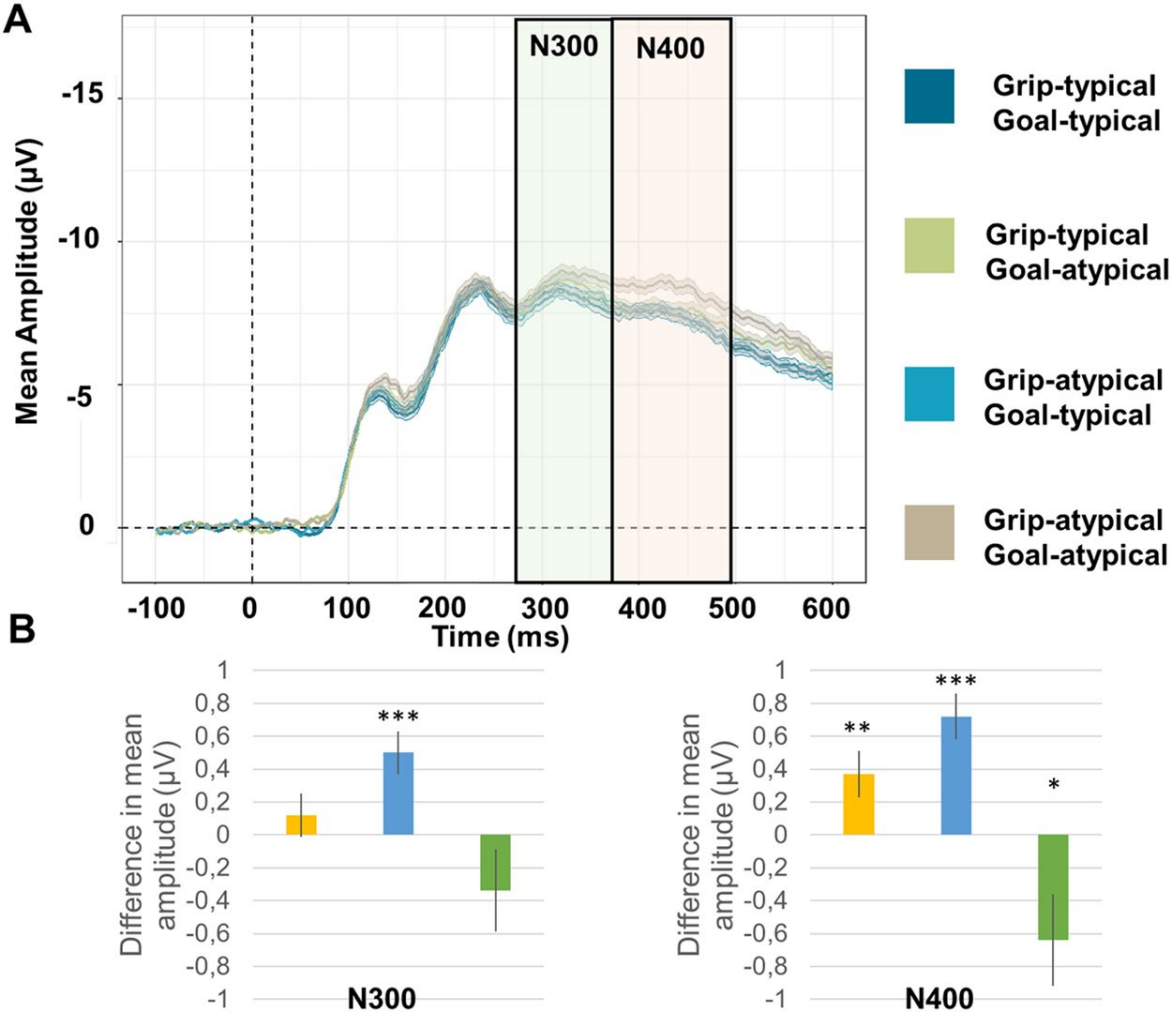
(**A**) ERP as a function of Grip typicality and Goal-typicality at the anterior site. The green font represents the time-window of the N300 component. The orange font represents the time-window of the N400 component. Ribbons represent standard errors. (**B**) Mean estimates of the main effect of grip-typicality (yellow bar), main effect of goal-typicality (blue bar) and Grip x Goal interaction (green bar), for the N300 and N400 components. Error bars represent standard errors. *p < 0.05; **p < 0.01; ***p < 0.001.

Analysis of the mean peak amplitude of the N300 component revealed a main effect of Goal-typicality, *F*_1,81_ = 15.11, *p*_*corrected*_ < 0.001, *Westfall’s d* = 0.15, but no main effect of Grip-typicality (*F*_1,81_ = 0.93, *p*_*uncorrected*_ = 0.338, *Westfall’s d* = 0.04). Atypical goals were more negative than typical goals (Goal-atypical – Goal-typical = 0.50 µV, *SE* = 0.13). Analysis of the mean peak amplitude of the N400 component revealed a main effect of Grip-typicality, *F*_1,81_ = 7.34, *p*_*corrected*_ = 0.016, *Westfall’s d* = 0.11, a main effect of Goal-typicality, *F*_1,81_ = 27.44, *p*_*corrected*_ < 0.001, *Westfall’s d* = 0.21 and a significant Grip-typicality x Goal-typicality interaction, *F*_1,81_ = 5.43, *p*_*corrected*_ = 0.045, *Westfall’s d* = 0.18. Interestingly, both main effects showed increased negativity for atypical conditions in comparison to typical conditions (Grip-atypical – Grip-typical = 0.37 µV, *SE* = 0.14; Goal-atypical – Goal-typical = 0.72 µV, *SE* = 0.14). Post-hoc tests indicated that the “Grip-atypical Goal-atypical” condition was more negative than the three other conditions, namely “Grip-atypical Goal-typical”, *t*_81_ = −3.56, *p* = 0.002, “Grip-typical Goal-atypical”, *t*_81_ = −5.35, *p* < 0.001 and “Grip-typical Goal-typical”, *t*_81_ = −5.62, *p* < 0.001, which were not significantly different from one another (all *p* > 0.176). Results are displayed on Fig. 2.

## Discussion

The current experiment examined the ERP components related to the identification of correct object-directed actions while carefully dissociating the role of static motor act components and goal-related information in action recognition. Object-directed action photographs could present grip and/or goal violations so that the two dimensions were manipulated independently. This design was appropriate to identify both the unique contribution of each dimension, and the integration of the two dimensions during the whole process of action recognition. Participants were asked to judge the overall correctness of object-directed actions such as pouring from a teapot or writing with a pencil, without any specific mention of grip and goal dimensions. Overall, results showed that the identification of object-directed actions induced P100, N170, P300, N300 and N400 components. The polarity and topography of these components were very similar to those previously reported for the processing of visual stimuli^31,33,39,42,43^. Action differences in terms of grip or goal visual information–including differences in terms of low-level features such as luminance, contrasts, gradients–were already visible on the P100/N170 components (“weak” effect sizes, 0.08 < *d* < 0.10). Independent post-perceptual processing of action goals was later observed on the anterior N300 component (“weak” effect sizes, *d* = 0.15), before the integration of grip and goal information on the N400 component (“weak” to “moderate” effect sizes, 0.11 < *d* < 0.21). The following section first discuss each effect separately, and subsequently integrate these effects with respect to the literature on action recognition.

The visual processing of grip configuration and action goal information was found to modulate both the P100 and the N170 components independently. The time windows of these two components are fairly congruent with previous “brain microstate” EEG studies on action processing, which identified periods of stability in the brain activity between roughly 0 and 120-ms over the visual cortex and between roughly 120 and 200-ms over the posterior temporal and inferior parietal cortices^16,17^. The P100 component is routinely used in ophthalmology to evaluate the integrity of the visual cortices^44,45^, and the N170 has been shown to be particularly sensitive to inversion effects (comparison between upright/upside-down pictures) of body parts and, to a lesser extent, of objects^42,46^. Both components have been linked to the activity of the visual cortices, the P100 being generated by the primary visual cortex, and the N170 by the associative visual cortices at the border of the temporal and parietal cortices^42^. Consequently, modulations of these components have been related to variations in the information contained in the visual stimuli. In our study, similar modulations of these components were induced by the presentation of different grip configurations on the one hand and different visual goals on the other hand. This suggests that the visual system is able, from the first steps of action processing, to visually differentiate photographs displaying typical grips from photographs displaying atypical grips (irrespective of the visual goal displayed) and photographs displaying typical goals from photograph displaying atypical goals (irrespective of the visual grip displayed). Previous studies have attributed such early brain modulations either to the processing of motor act components^17^ or to the processing of action goals^35^. The present experiment suggests that both dimensions contribute to these early modulations in an independent manner. It is not surprising that variations of grip configurations or visual goals in the picture stimuli induce modulation of early EPR components, like any perceptual difference (shape, colour, etc.) would do^13^. Nonetheless, it is important to note that visual differences related to grip and goal dimensions are equally noticed by the observers.

The N400 component was found modulated by the interaction between the grip configuration and the action goal information. We found that the N400 generated when both the action-goal and the grip-configuration were atypical was more negative than the one generated by any of the remaining combinations. In spite of very different design and stimuli, this pattern has been previously reported by Bach *et al*.^31^. In their study, participants had to evaluate whether two objects could be inserted together. Objects were sequentially presented and could have a correct functional relationship or not (screwdriver and screw vs screwdriver and keyhole), or a correct motor relationship or not (horizontal screwdriver and horizontal screw). They found a more negative N400 when two semantic violations (i.e., in terms of functional relationship between the two objects on the one hand, and in terms of motor relationship between the two objects on the other hand) were present in the action (e.g., a horizontal screwdriver and a keyhole in a vertical orientation) than in any of the remaining combinations. In contrast, in spite of very similar design and stimuli, Chang *et al*.^33^ did not found the expected modulation of the N400 component as a function of action typicality. In their study, fully typical object-directed actions were compared to fully atypical object-directed actions (i.e., on both the action goal and grip configuration dimensions; e.g., an upside-down pencil with a power grasp). They argued that the absence of N400 sensitivity to semantic violation during action processing was due to participants’ inability to “*rapidly match the semantic information conceptually*” (p. 7). Another possibility is that the N400 is modulated by the importance of semantic integration performed by the participants on the stimuli and that the limited combinations of grips and goals in their experiment may have reduced the semantic integration requirement. Consistent with this idea, the N400 component has been proposed to reflect a neurocognitive mechanism involved in the construction of meaning^30,47^ and not the mere reflection of stimulus recognition^47^. In both our study and Bach *et al*.^31^ study, action stimuli varied along several combinations of grips and goals that needed to be integrated to perform the recognition task and N400 modulations were observed. Therefore, these findings corroborate the interpretation of the N400 as a marker of semantic construction and integration across different domains such as action and language processing^30,48^.

Interestingly, the processing of the visual actions generated an N300 component which was modulated by the typicality of the action goal, but not the typicality of the action grip. What lies behind N300 components is unclear. Sometimes, it has been interpreted as an extension of the N400 components with similar sensitivity^25,29^ and sometimes as being a component clearly distinct from the N400^49^. Our results suggest that the N300 is, at least partially, independent from the N400 component. Chang *et al*.^33^ reported an N300 with a posterior distribution. Fully typical actions were more negative than fully atypical actions. Their N300 component was very similar (in terms of topography and functional sensitivity) to the “Recognition potential”^27^. Thus, they proposed that the increased negativity for the fully typical actions reflected an easier access to visual semantic memory in comparison to fully atypical actions. However, this interpretation does not stand for our N300, as both the topography and functional sensitivity do not fit. One may argue that our N300 sensitivity to goal-typicality could be simply driven by mere low-level perceptual differences between goal-typical and goal-atypical actions, as a similar sensitivity was found on the P100/N170 components. We believe that this is relatively unlikely, however. If goal-typicality effects had their roots in mere low-level perceptual differences, they should be observed on each component identified, but they were not found on the P300 component. Thus, we argue that the sensitivity of our N300 to goal-typicality more likely reflects the processing of goal-related information at a “post-perceptual” stage of action processing (i.e. accessing a representation of the action goal), even though the exact cognitive mechanism remains to be identified.

Taken as a whole, our results suggest that observers equally detect low-level visual differences between typical versus atypical stimuli for grip and goal dimensions respectively, reflected by the P100/N170 modulations. In addition, at a later “post-perceptual” stage of action processing, we also found that the access to a representation of the action goal precede the access to a representation of the grip configuration, as goal-related modulations were already visible on the N300 component, before the grip-related modulations only observed later on the N400 component. The integration of the two dimensions was also observed on the N400 component. Therefore, it may be possible that the post-perceptual processing of action goal may participate to the semantic processing of grip configuration. Many sources of evidence have extensively highlighted the importance of goals in action recognition^5–7,50–53^. The present experiment further demonstrates that goals are not only important overall, but that this importance arises early and before other action components during the visual processing of others’ actions. It now remains to establish whether the use of static action stimuli, necessary for the current investigation, could have limited the influence of grip information and relatively made the early contribution of goal-related information more salient. Both static and dynamic components are involved in the processing of motor acts^54,55^ but the generalisation of the findings to dynamic action stimuli should be addressed in the future.

Among the techniques available for cognitive researchers, EEG is a powerful tool to investigate dynamic cognitive phenomena, even when the stimulus to process is not dynamic. Action recognition tends to be more and more considered as a heterogeneous set of various dynamic mechanisms rather than a unitary process^56^ and would rely on both specific and domain-general abilities^36,57^. In parallel, EEG is increasingly recommended to investigate the commonalities and differences between action and language processing^30,48^. Therefore, we believe that the contribution of EEG to the understanding of action recognition will grow in the future. Our study showed that visual differences in terms of grip configuration and action goal can be detected from the first stages of action perception and that the post-perceptual processing of the two action dimensions follows different time courses. ERP correlates of visual action processing highlight the first role of goal-related information in the comprehension of object-directed actions. Our study points to several ERP components that may be related to different processing steps in the recognition of complex goal-directed actions. Future research may want to focus on how the dynamics of action processing may adapt to different individual characteristics and task contexts. The present findings may provide important directions in this regard.

## Methods

### Participants

Thirty-one participants took part in the study. Three participants were excluded because of excessive noise in the EEG signal. The twenty-eight remaining participants (mean age 21, range 18–29, 10 males) were all right-handed (handedness quotients range 27–100%, mean 83%^58^,), and reported normal or corrected-to-normal vision. They provided written informed consent and received twenty euros for their participation. The protocol was approved by the Ethical Committee of the University of Lille and was in accordance with the declaration of Helsinki (1964, revised in 2013).

### Stimuli

**Figure 3:**
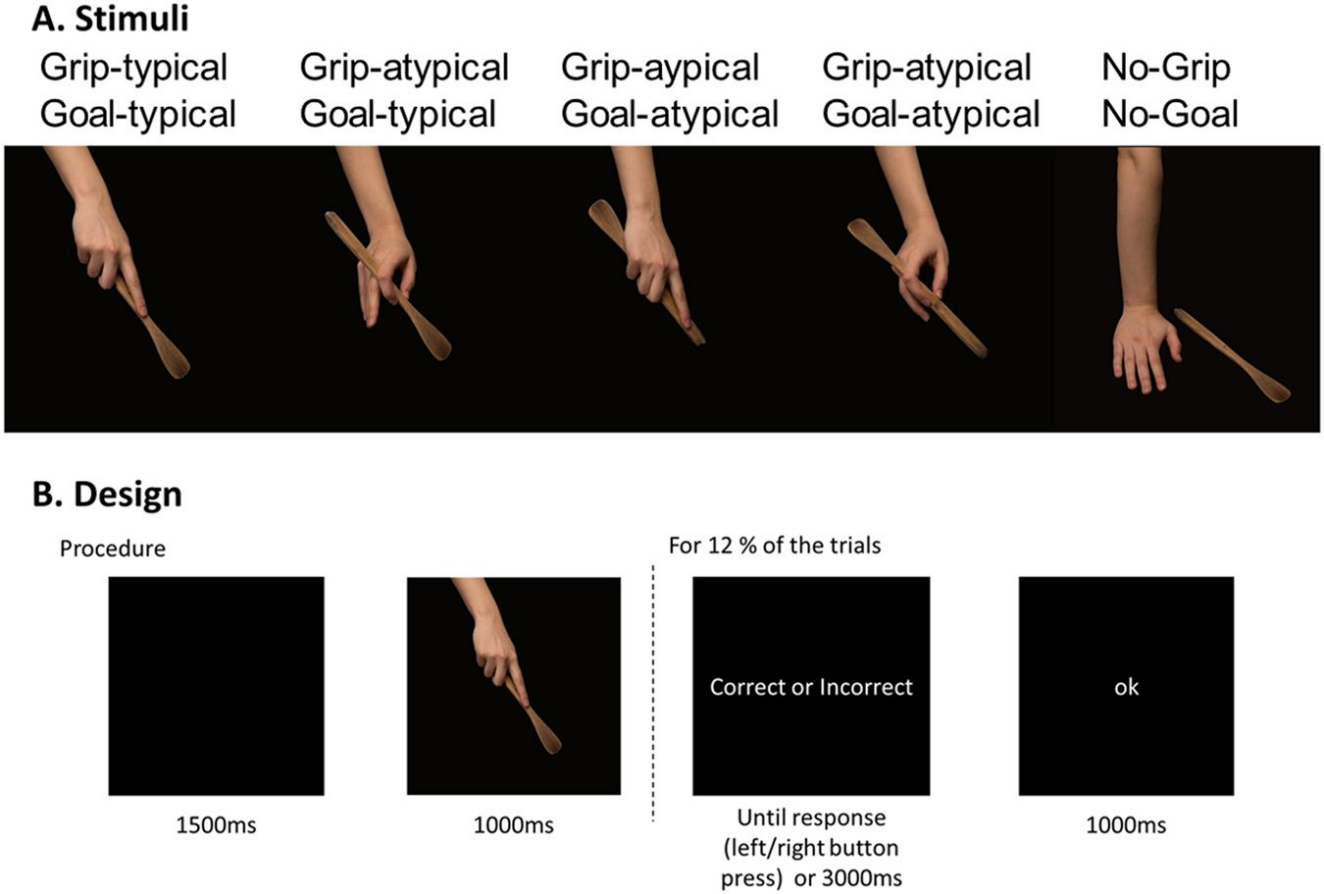
Design and procedure of the experiment. (**A**) Stimuli were divided in four experimental conditions by manipulating the typicality of the grip and the goal of the action. A fifth neutral condition was added as a control of object information. (**B**) Procedure on a given trial. Responses were prompted for only 12% of the trials.

Twenty objects were selected. For each reference object, five coloured 1024 ×683 pixels photographs were taken, all displaying a hand and a tool-object. Four out of the five pictures of the set presented hand-on-object actions. The remaining photograph corresponded to a no-action picture showing the hand and the object without any interaction between them. Actions were always performed with the right-hand by the same right-handed actress and photographs were framed in such a way that only the forearm, the right hand and the object were visible. Photographs always included the tool-object but never the recipient object on which the tool acts on (e.g., a nail for an action with a hammer). All information outside of the hand and tool-object that could influence action processing was eliminated in order to present object-related actions in a context as neutral as possible. An example of the stimuli can be found in Fig. 3A. The full set of stimuli is available as Supplementary Materials.

For each reference object, actions could be typical or not along the grip dimension and/or the goal dimension. Grips applied on the object could be typical or not according to the typical manipulation of the object. For instance, a precision grip applied to a pencil is typical, whereas a power grasp is not. The typical goal could be achieved or not according to the typical function of the object. For example, a pencil in upright position allows one to write (typical goal possible), whereas a pencil upside-down does not (typical goal impossible). Importantly, the typical goal could still be achieved even when the grip was atypical, and vice-versa. Thus, grip and goal dimensions were manipulated independently. Importantly, object identity was equally diagnostic of goal and grip typicality. Recognising typical goal required both to retrieve the object identity and to process the position of the object relative to the hand. Recognising typical grip required both to retrieve the object identity and to process the grip configuration of the hand. Therefore, although both grip and goal typicality relied on object identity, the position of the object relative to the hand was not informative of grip typicality. Similarly, the grip configuration of the hand was not informative of goal typicality.

The effect of grip and goal typicality on the perception of the overall correctness of the action was verified in a pre-test. For each action photograph, nine participants who were not included in the EEG experiment were asked to determine whether the action was correct or not according to the typical use of the object. The word “use” (“utilisation” in French) was chosen as it addresses both the visual kinematic component (i.e., “how to use the object”) and the goal component (i.e., “why to use the object”). Participants were able to successfully classify correct (i.e. with both typical grip and goal) and incorrect (with either or both atypical grip and goal) photographs (mean accuracy = 90% +/− 10 *SE*). A Chi-square test for independence indicated that performance was equally distributed between conditions [χ^2^_3_ = 0.55, *p* = 0.907]. Participants were thus able to recognise correct and incorrect action photographs. Importantly, results from the pre-test confirmed that participants took into account both dimensions when judging the overall correctness of the action, as photographs in which only one of the two dimensions was atypical were adequately judged as incorrect (i.e., Grip typical but Goal atypical or Grip atypical but Goal typical).

To sum up, the 100 picture stimuli were divided in 5 conditions: “Goal-typical Grip-typical”, “Goal-atypical Grip-typical”, “Goal-typical Grip-atypical”, “Goal-atypical Grip-atypical”, “No-goal No-grip” (goal and grip were then neither typical nor atypical). This “neutral” condition was first included as a possible control condition in order to estimate the contribution of mere object processing to action recognition. Since “neutral” no action pictures were processed very differently from the action pictures, this condition was finally not included in the analysis.

### Procedure

Participants first provided written informed consent. They were then seated in a dimly illuminated room in front of a computer screen (1024 × 768, 60 Hz) and the EEG cap was positioned. Participants were carefully instructed to avoid eye and body movements during the recording session. Then, the experiment could start.

Each trial started with a black screen of 1500-ms followed by the object-related action photograph for 1000-ms. They were instructed to evaluate, for each action, whether the overall action was correct or not. They were explicitly told that a correct action corresponded to the typical use of the object. Moreover, they were asked to provide an explicit behavioural response only when a response mapping display followed the action picture. Behavioural responses were prompted on 12% of the trials and participants did not know the response mapping in advance. On those “response” trials, the photograph was followed by a screen on which the words “correct” and “incorrect” were written on each side of the screen. The left/right position of the correct/incorrect responses was counterbalanced across trials so that participants could not prepare their motor response while processing the action photograph. This choice was made to avoid contamination of the relevant EEG signal by motor preparation. The response screen remained visible until the participant’s response or for 3000-ms. Participants responded on two separate keys of a response box with their left and right thumbs for correct versus incorrect. After their response, a black screen with “ok” was displayed for 1000-ms. The “ok” screen was only for participants to know that their answer had been taken into account, but was not informative about the accuracy of their answer. The “ok” screen further allowed to avoid introducing variation on trial duration due to variation in participant’s response, as the beginning of the next trial was not dependent of the participant’s response time. Each trial was repeated 6 times, in 6 different blocks. Overall, there was 20 objects × 5 conditions × 6 repetitions = 600 trials. Consequently, there was a maximal of 120 trials per condition per participant. All conditions and objects were equally represented in each block. Trials inside blocks and blocks were randomly presented. The design is presented in Fig. 3B. Blocks were about 7-minute each and breaks were proposed between blocks.

A training session involving twelve representative trials with three objects not included in the experimental session was performed beforehand. In contrast to the experimental phase, a feedback on the accuracy of the response was provided to the participant during the training session. The overall experiment lasted about 2-hour. The experiment was conducted with E-Prime V2.0.10.353 software (Psychology Software Tools, Pittsburgh, PA).

### EEG recording and analysis

EEG data were continuously collected from 128-channel Biosemi ActiveTwo (Biosemi B.V., Amsterdam, Netherlands) at a sampling rate of 1024 Hz thanks to ActiView software. Electrode caps covering the whole head with equidistant-layout were used. Electrode offset was kept below 20 µV. The offset values were the voltage difference between each electrode and the CMS-DRL reference. Electrooculographic (EOG) activities were recorded bipolarly using electrodes placed near both canthi (for measuring horizontal eye movements), and below and above the left eye (for measuring vertical eye movements, i.e., blinks). Four additional electrodes were placed above the flexor pollicis brevis of each hand to monitor the electromyographic activity of the thumb (two on the right hand, two on the left hand). The electromyographic data were not directly related to the present paper, and thus will not be discussed any further. A last electrode was placed on the left mastoid. Offline analysis was performed using BrainVision Analyzer 2.1 (Brain Products GmbH, Munich, Germany). One electrode (D7) did not register the brain activity for all subjects and was thus interpolated. This electrode was not considered in the following analysis. Its interpolation could then not affect the results. Eye movements artefacts were first corrected using the Gratton and Coles’ method^59,60^. Remaining artefacts on the signal were marked manually by visual inspection on the continuous recorded EEG signal, regardless of the conditions. The raw signal was then filtered using a high pass filter at 0.1 Hz (zero-phase shift Butterworth filter, order 2) and a low pass filter at 100 Hz (zero-phase shift Butterworth filter, order 4). The continuous EEG signal was re-referenced on average reference. The left mastoid was considered as a reference but could not be used because of excessive noise in the mastoid signal. The signal was then segmented into 1200-ms periods (200-ms before the action photograph onset, 1000-ms after action photograph onset). At this point, epochs contaminated by artefacts were not considered anymore. About 15% of the trials were removed for the following analyses (Mean +/− *SD* remaining trials per participant: “Goal-typical Grip-typical”, mean 103 +/− 9 trials; “Goal-atypical Grip-typical”, mean 104 +/− 8 trials; “Goal-typical Grip-atypical”, mean 103 +/− 8 trials; “Goal-atypical Grip-atypical”, mean 101 +/− 9 trials; “No-goal No-grip”, 100 +/− 10 trials). Baseline correction was applied using the 200-ms time-window pre-action photograph onset. Finally, the EEG signal was averaged across trials for each condition.

**Figure 4:**
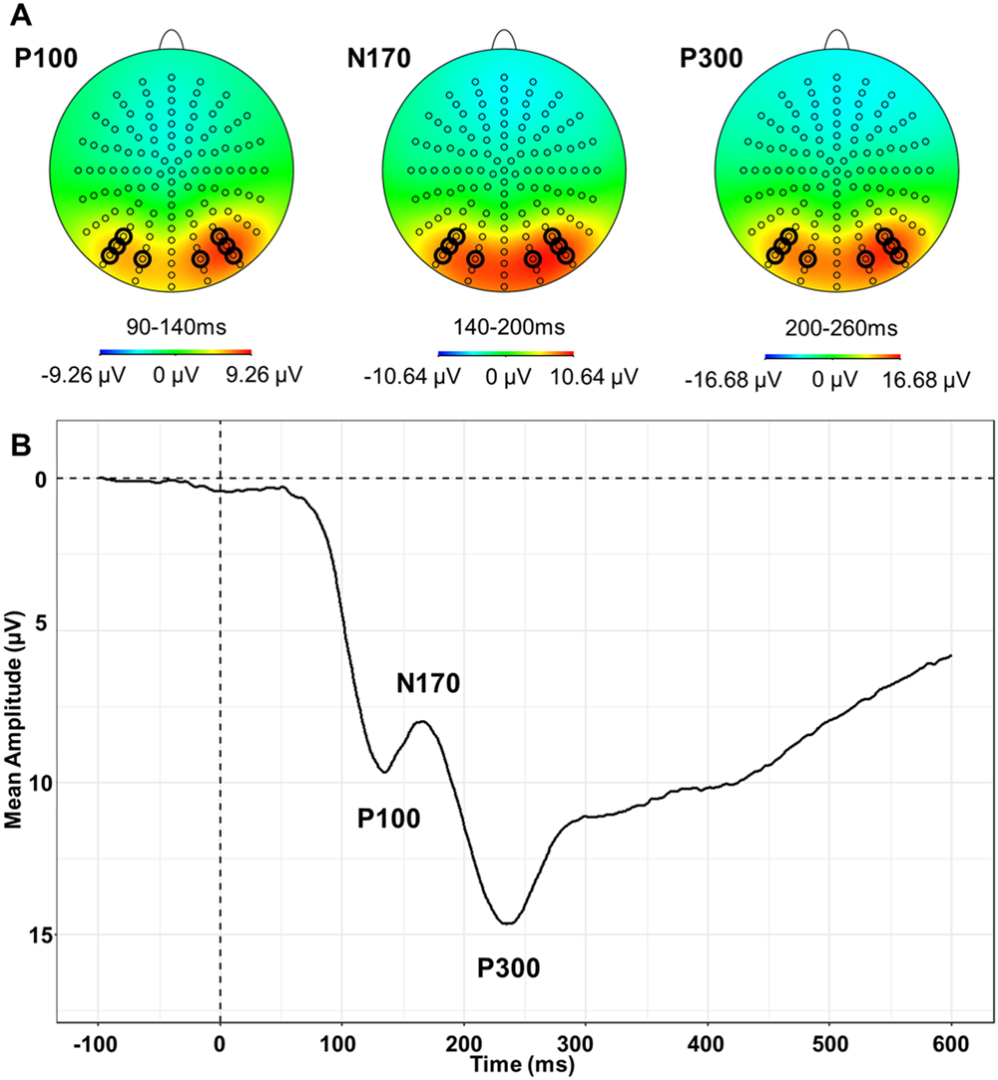
(**A**) Scalp map distribution corresponding to the two identified time-regions. Bold circles indicate the electrodes that have been averaged to obtain the mean amplitude of the P100, N170 and P300 respectively. (**B**) Grand average ERP at the posterior site.

**Figure 5:**
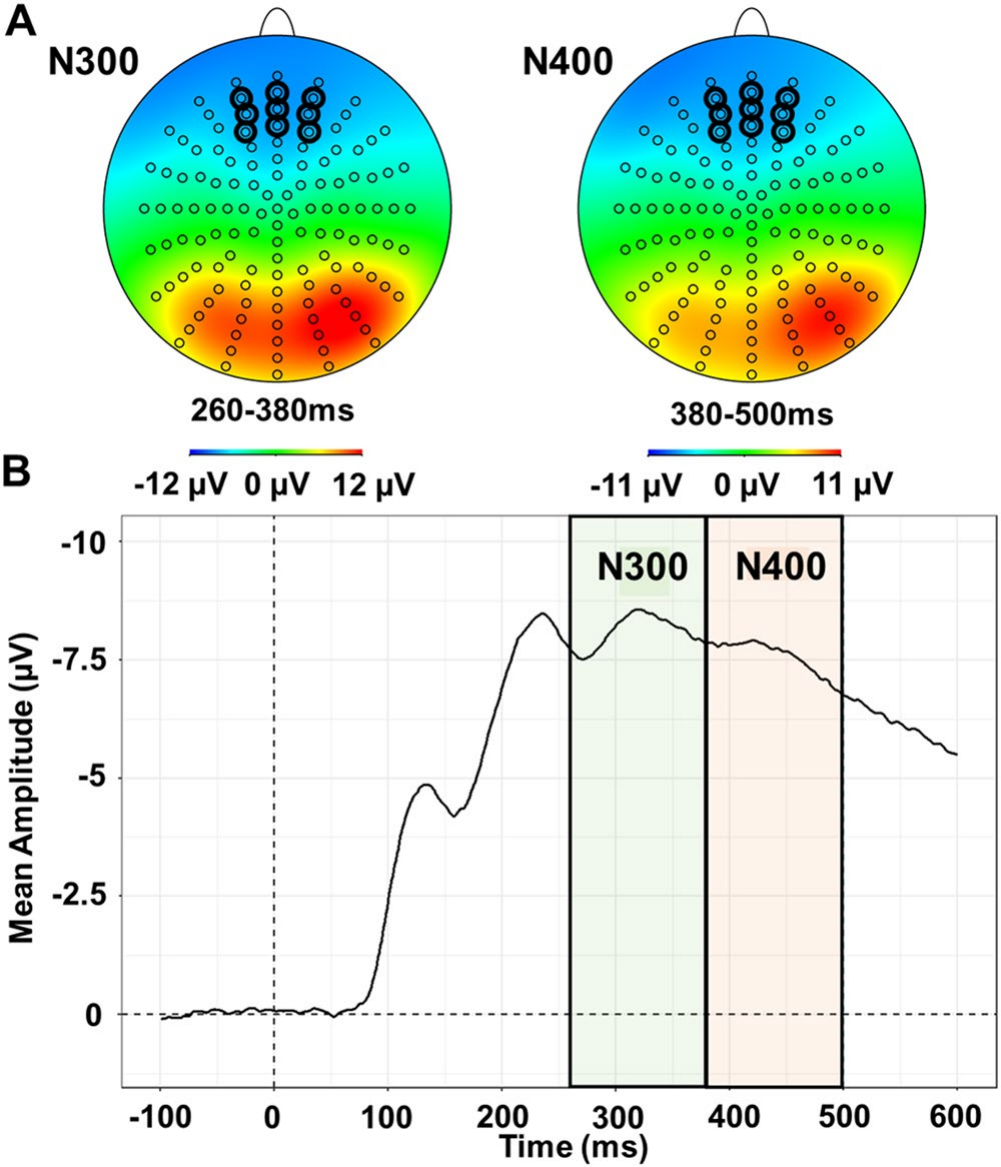
(**A**) Scalp map distribution corresponding to the two identified time-regions. Bold circles indicate the electrodes that have been averaged to obtain the mean amplitude of the N300 and N400 respectively. (**B**) Grand average ERP at the anterior site.

ERPs were averaged across all subjects and all conditions to define the analysis parameters^61^. Five ERP components were identified on the collapsed waveforms: P100 (90–140-ms), N170 (140–200-ms), P300 (200–260-ms), N300 (260–380-ms) and N400 (380–500-ms). Scalp map distributions were used to gather neighbouring electrodes that show the greatest activity for each component^39,62,63^. ERPs were collapsed across B6 – B7 – B8 – A28, and across A9 – A10 – A11 – A15 to represent maximal posterior right and left activity respectively for P100, N170 and P300^39,42,43,62–67^. ERPs were collapsed across C28 – C27 – C26 – C18 – C19 – C20 – C15 – C14 – C13 to represent maximal anterior central activity for N300 and N400^30,31,33,39,62,68^. Scalp map distributions and corresponding grand average ERP for the collapsed electrodes are presented in Fig. 4 for posterior site and Fig. 5 for anterior site.

Mean peak amplitudes and peak latencies (when available) were used as dependent variables. In order to best capture individual variability, mean peak amplitudes for the P100, N170 and P300 components were obtained for each participant by averaging the EEG activity on a +/−10-ms time-window around each individual maximum peak for each component and condition^39^. Peak latencies were obtained using the timing of the maximum peak for the P100, N170 and P300 components respectively for each individual and each condition. The identification of individual peaks for the N300 and N400 components was not always evident, as it is usually the case for late components. Mean peak amplitudes for these components were then obtained by averaging the activity over each time-window (260–380-ms for the N300, and 380–500-ms for the N400). As a consequence, peak latencies were not analysed for the N300 and N400 components.

### Statistical approach

Mean peak amplitudes/peak latencies were analysed using mixed-effect models to consider participants as a source of variation. Models then included grip-typicality, goal-typicality and the interaction between the two factors as fixed effects, and participants as random intercepts. Models were fitted with REML using the lmer function from “lme4 1.1-17” package^69^. Main effects and interaction were evaluated with the *F* statistics using the anova function of the “lmerTest 3.0-1” package^70^. This package allows to approximate the degree of freedom of the denominator using the Satterthwaite’s method, which has proven to produce acceptable type 1 error rates^71^. Post-hoc analyses were carried out using the “emmeans 1.3.4” package^72^ with Tukey’s method adjustment for multiple comparisons. Effect sizes were computed using the *Cohen’s d* adapted for mixed-effect models, hereafter “*Westfall’s d*”^73–75^. *Westfall’s d* is computed by dividing the difference of estimated means by the square root of the sums of the variance of the random parameters (i.e., the random intercept of participants and the residuals in our models). Bonferroni corrections were applied on the *F* statistics to account for the multiple analyses of the same brain regions: three times for the posterior site (P100, N170 and P300) and two times for the anterior site (N300 and N400).

## Supplementary information

Full set of stimuli.
