# Supplementary material for: Neural dynamics of grip and goal integration during the processing of others’ actions with objects: An ERP study
Source: Sci Rep. 2020 Mar 19;10:5065. doi: 10.1038/s41598-020-61963-7 (PMC7081278; doi:10.1038/s41598-020-61963-7)

Please find in the following pages each of the stimuli used in the experiment. For sake of simplicity, in the present document pages will always organized as followed :

Top left<br>Grip-typical<br>Goal-typical	Top right<br>Grip-typical<br>Goal-atypical
Bottom left<br>Grip-atypical<br>Goal-typical	Bottom right<br>Grip-atypical<br>Goal-atypical

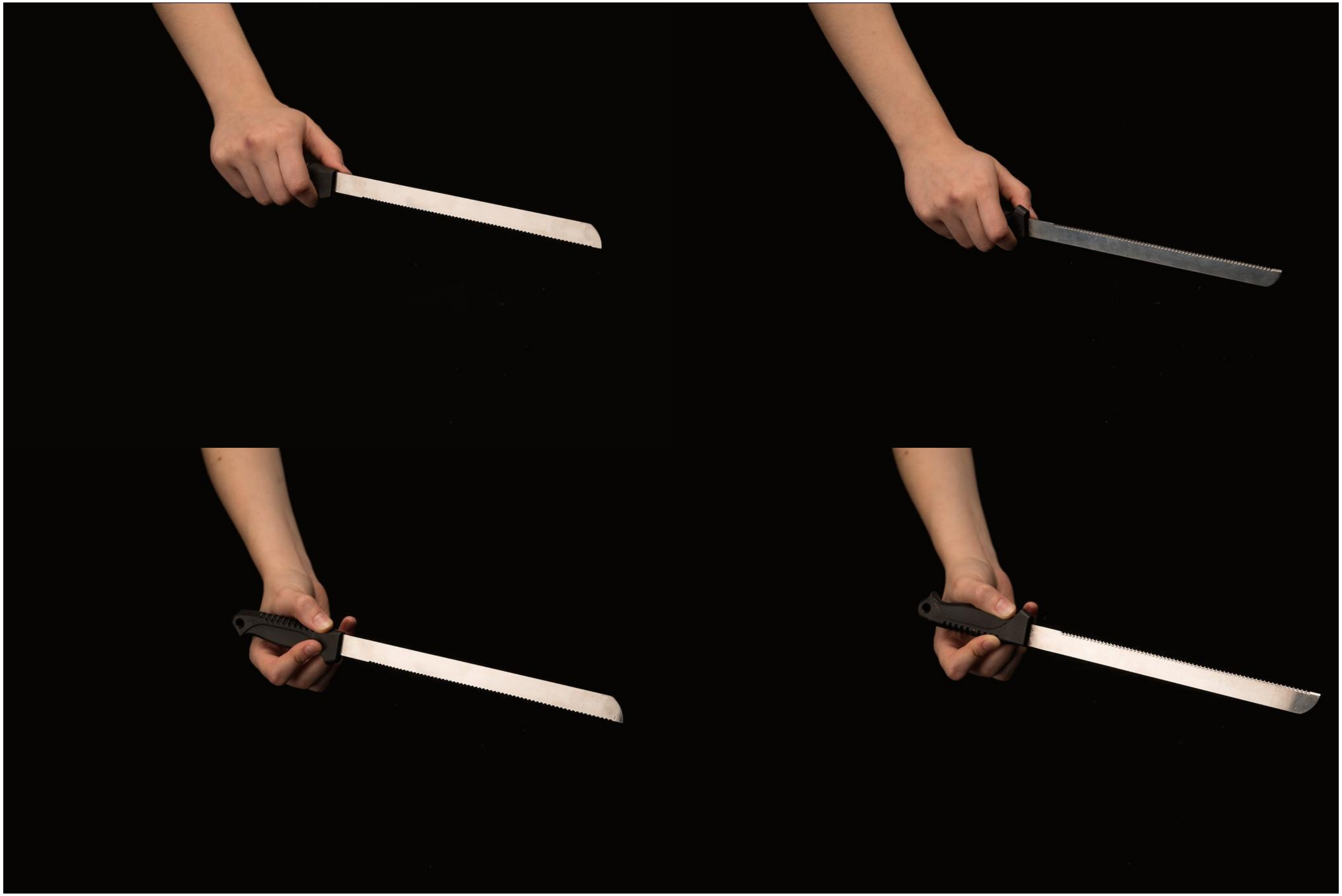

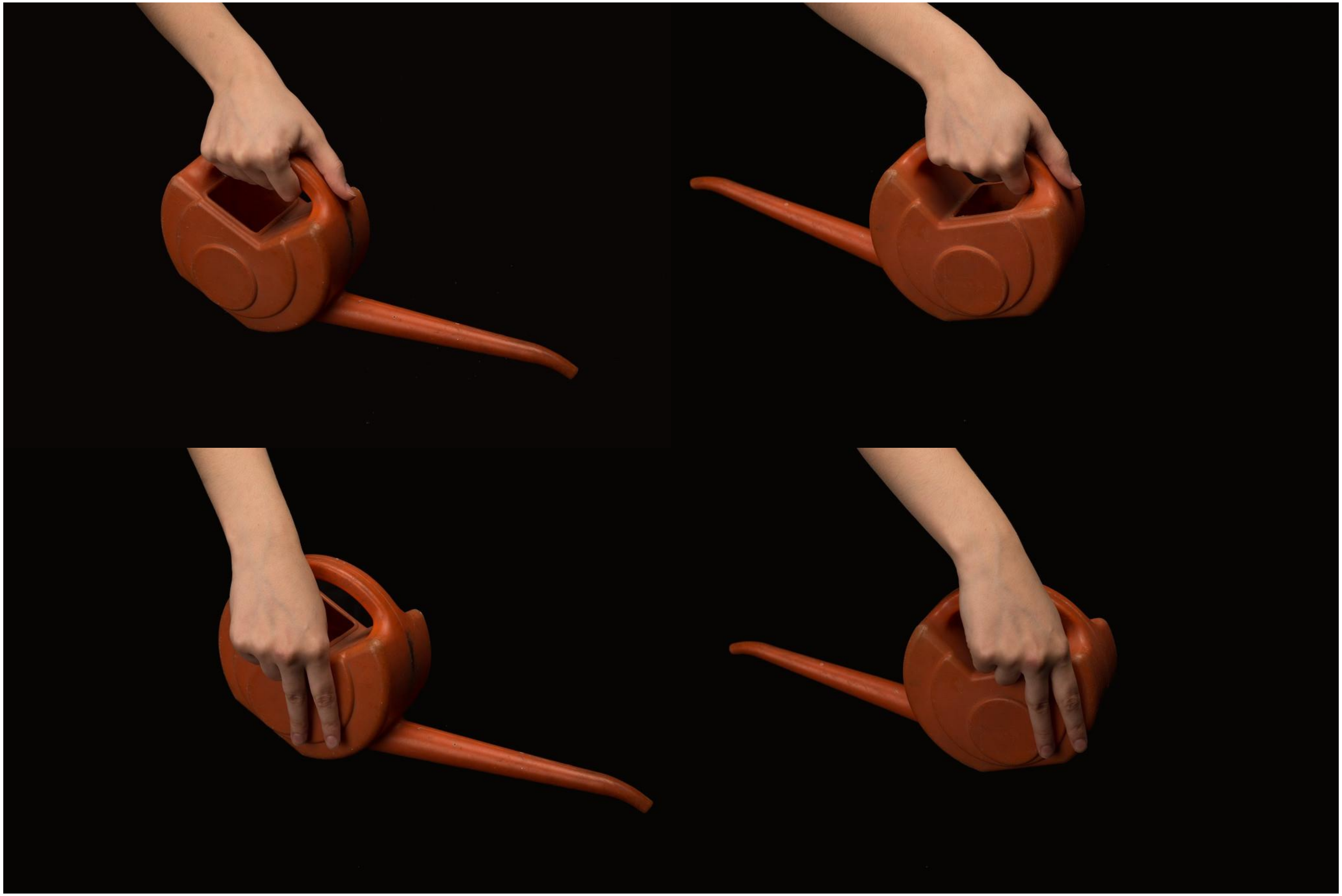

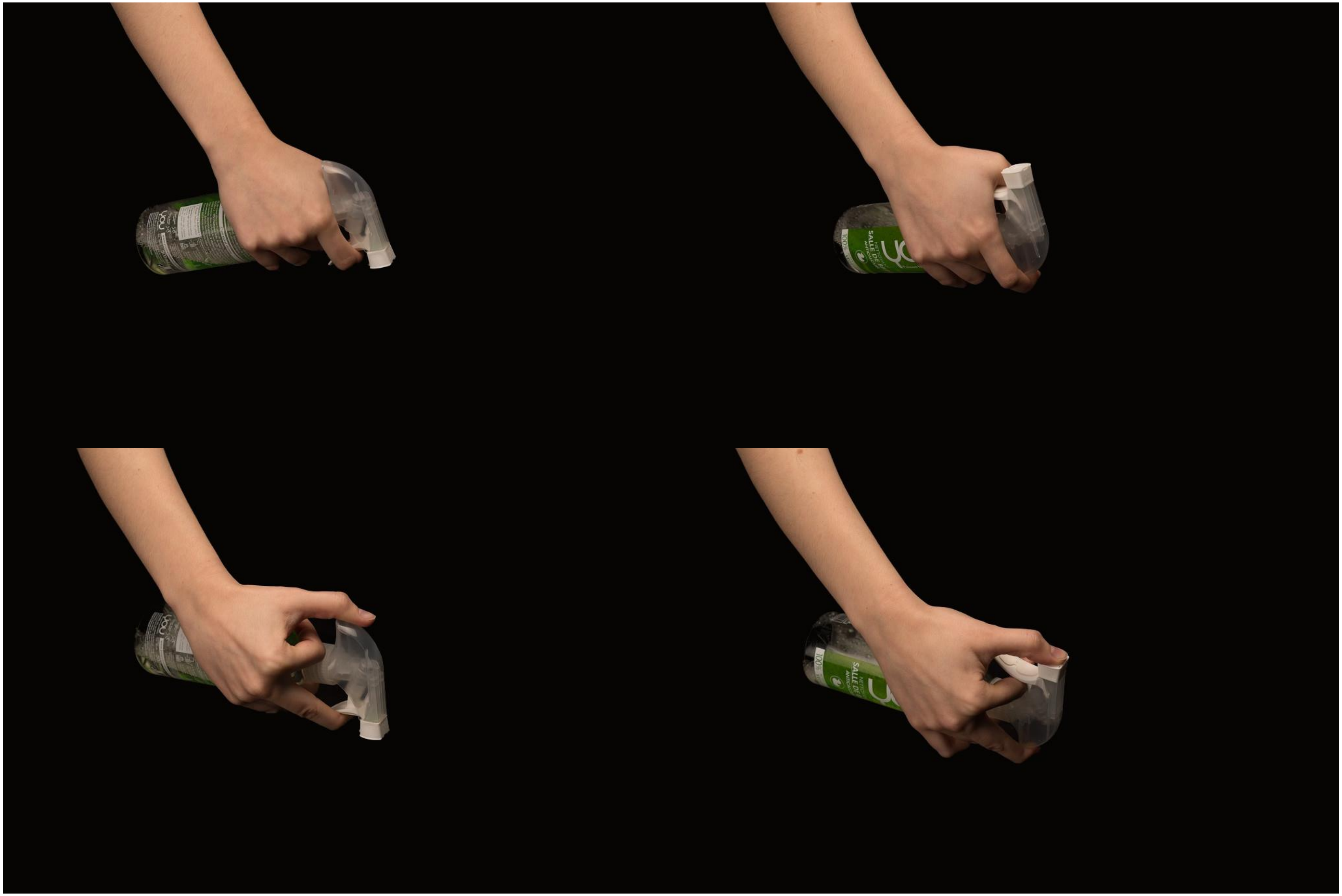

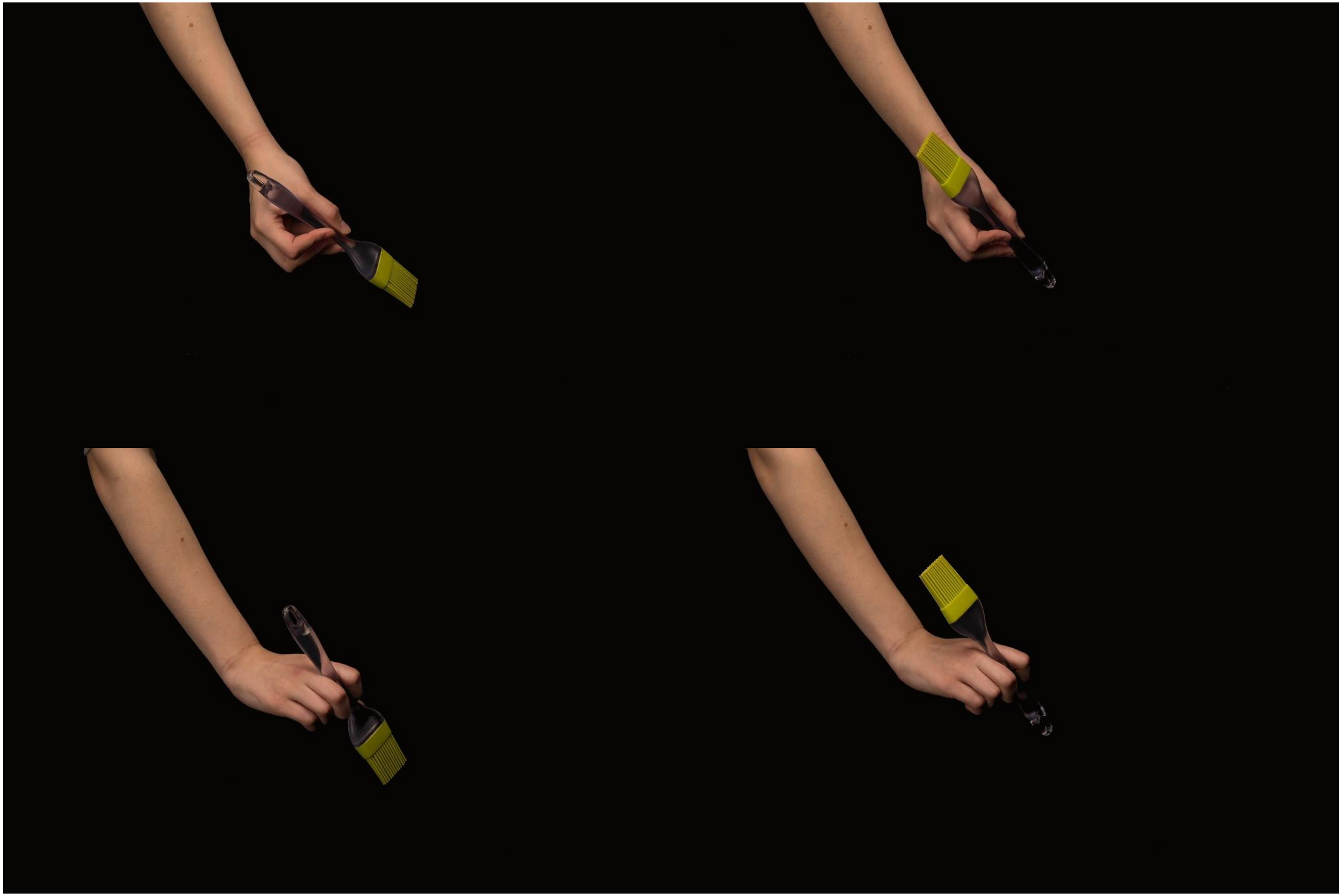

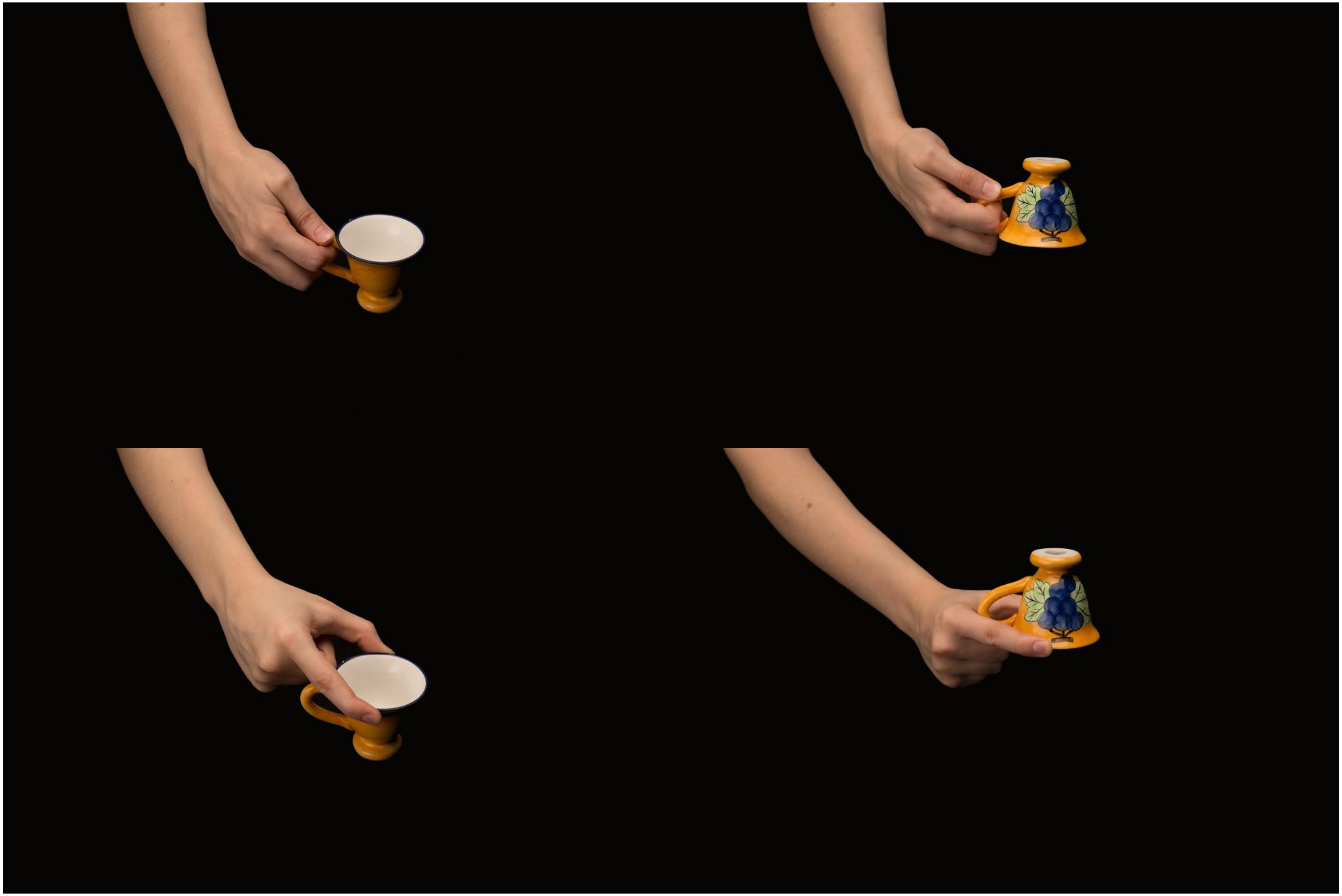

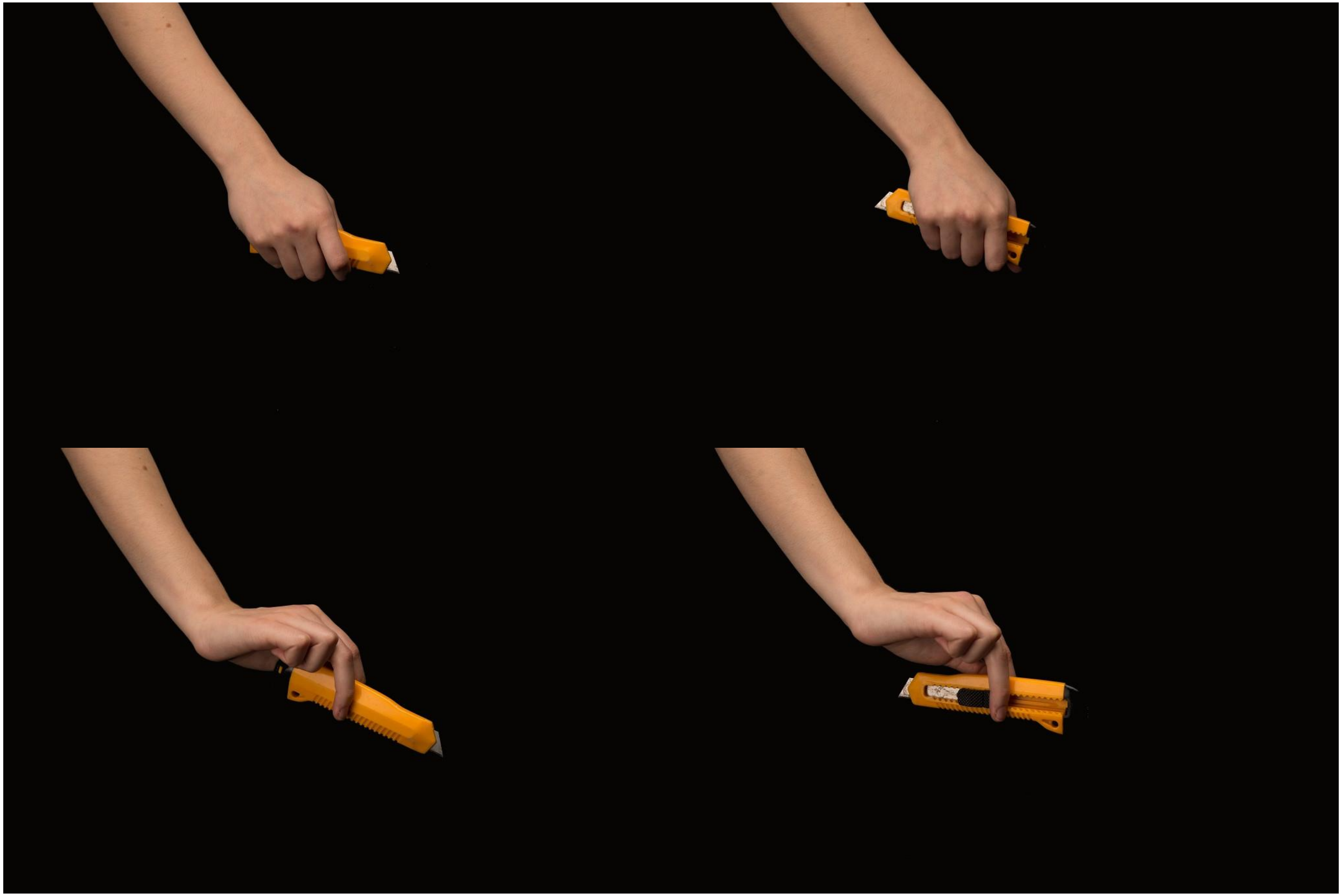

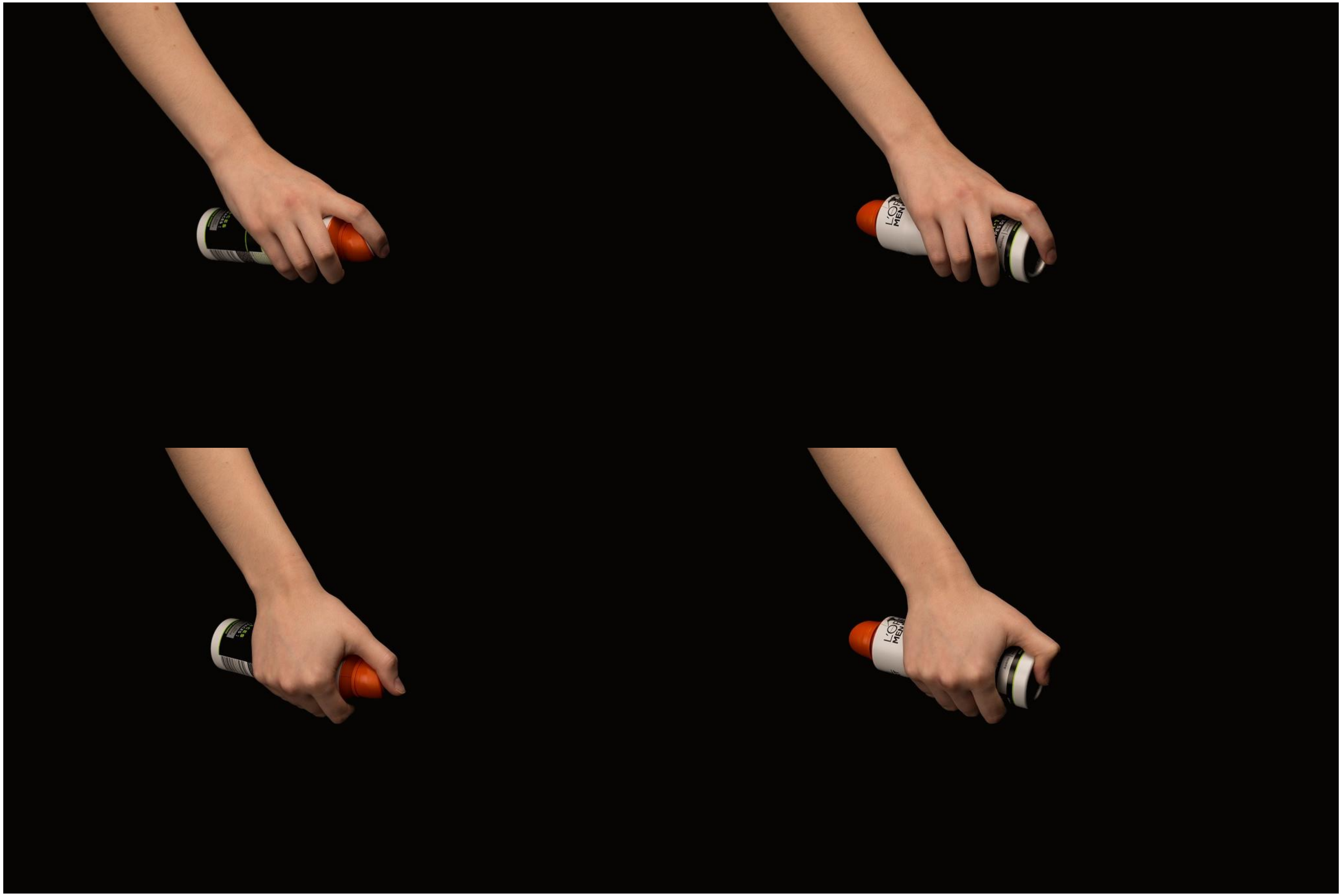

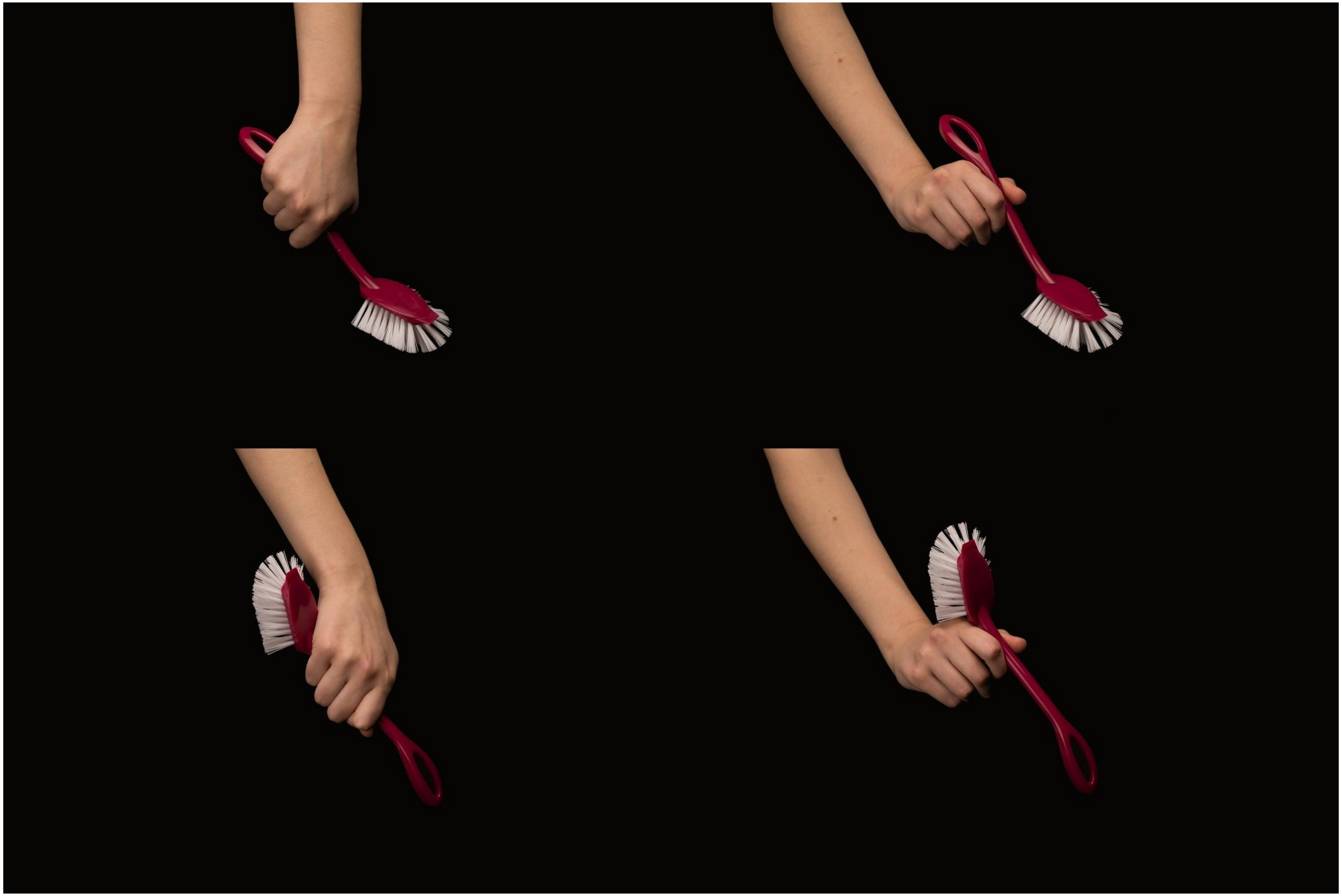

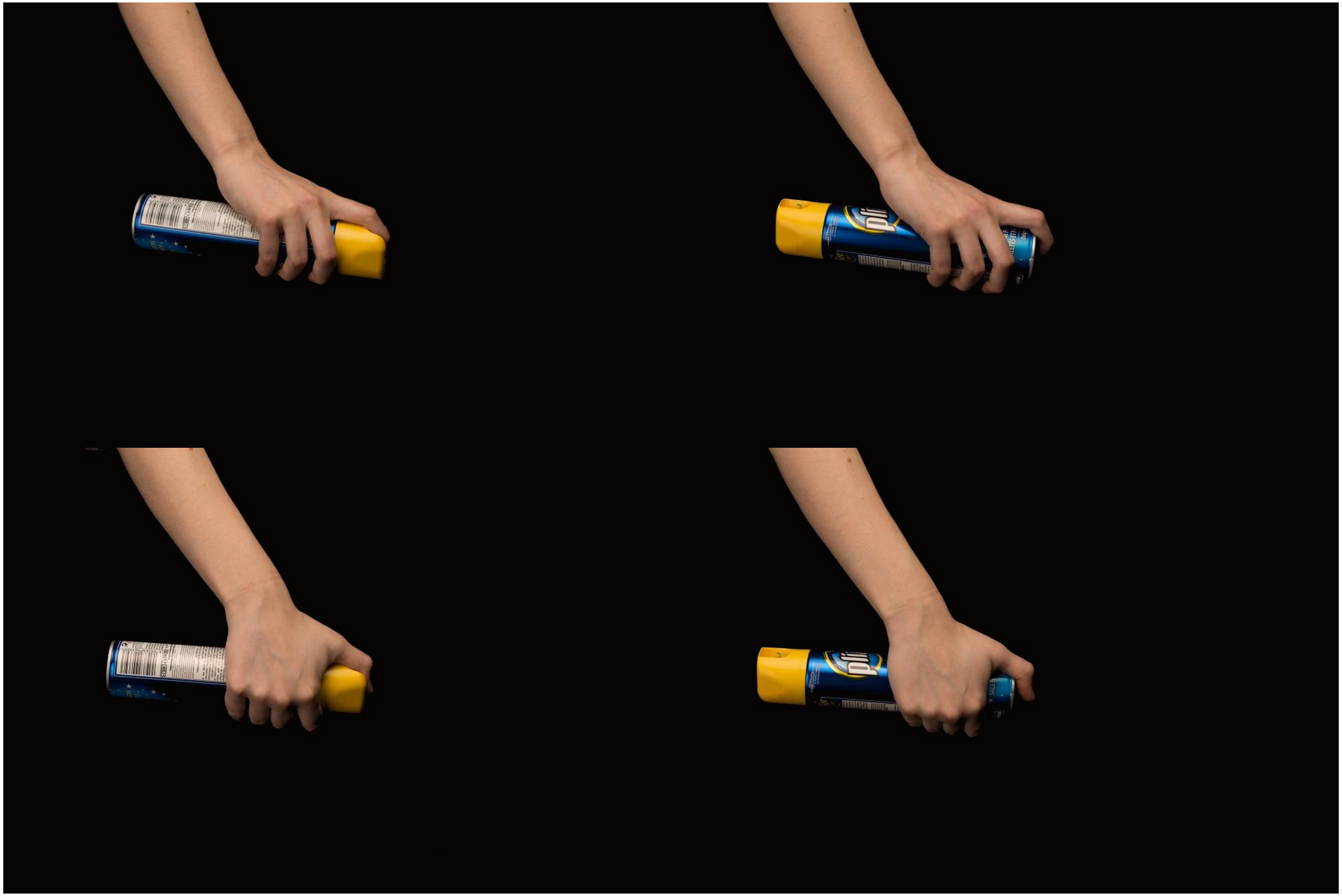

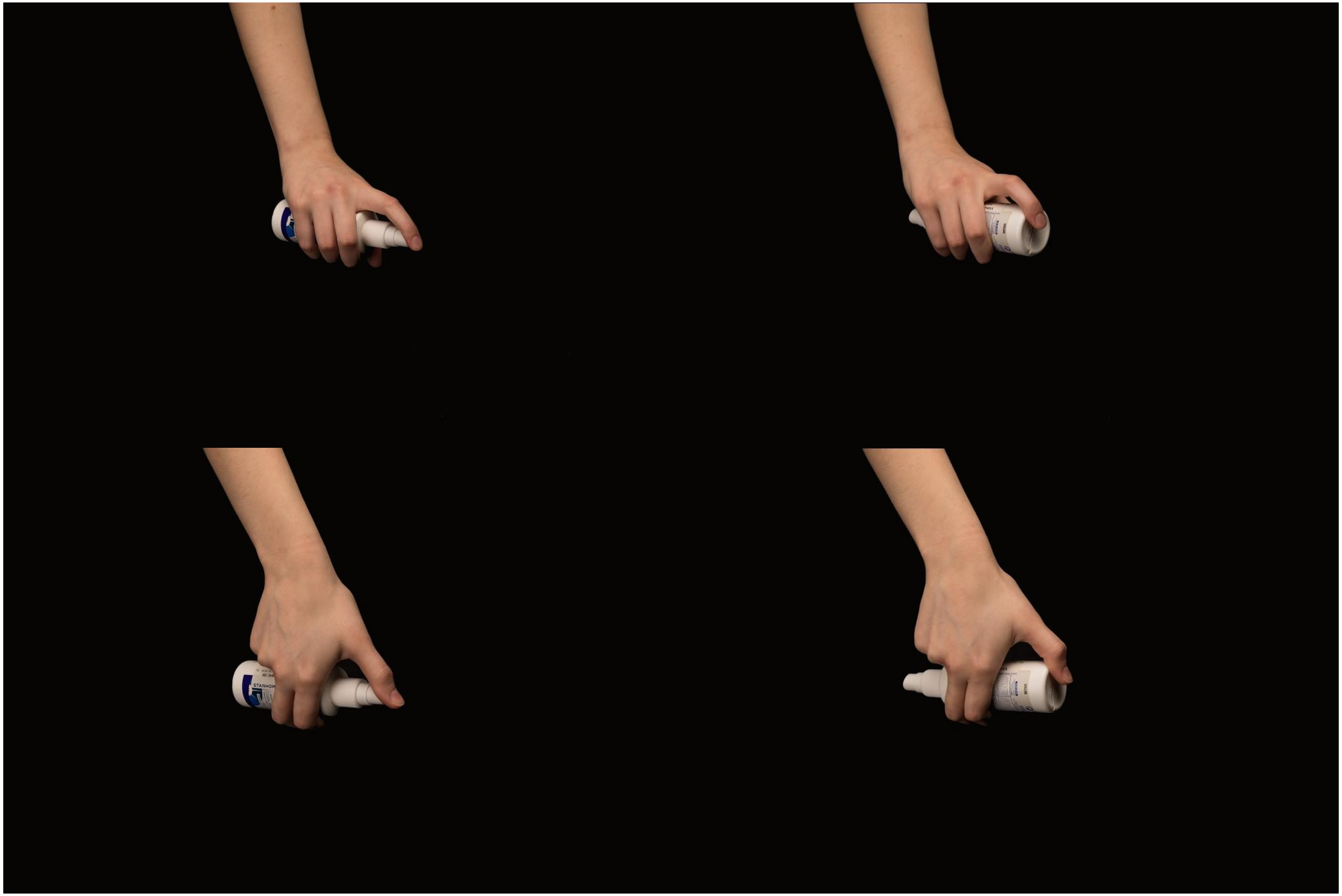

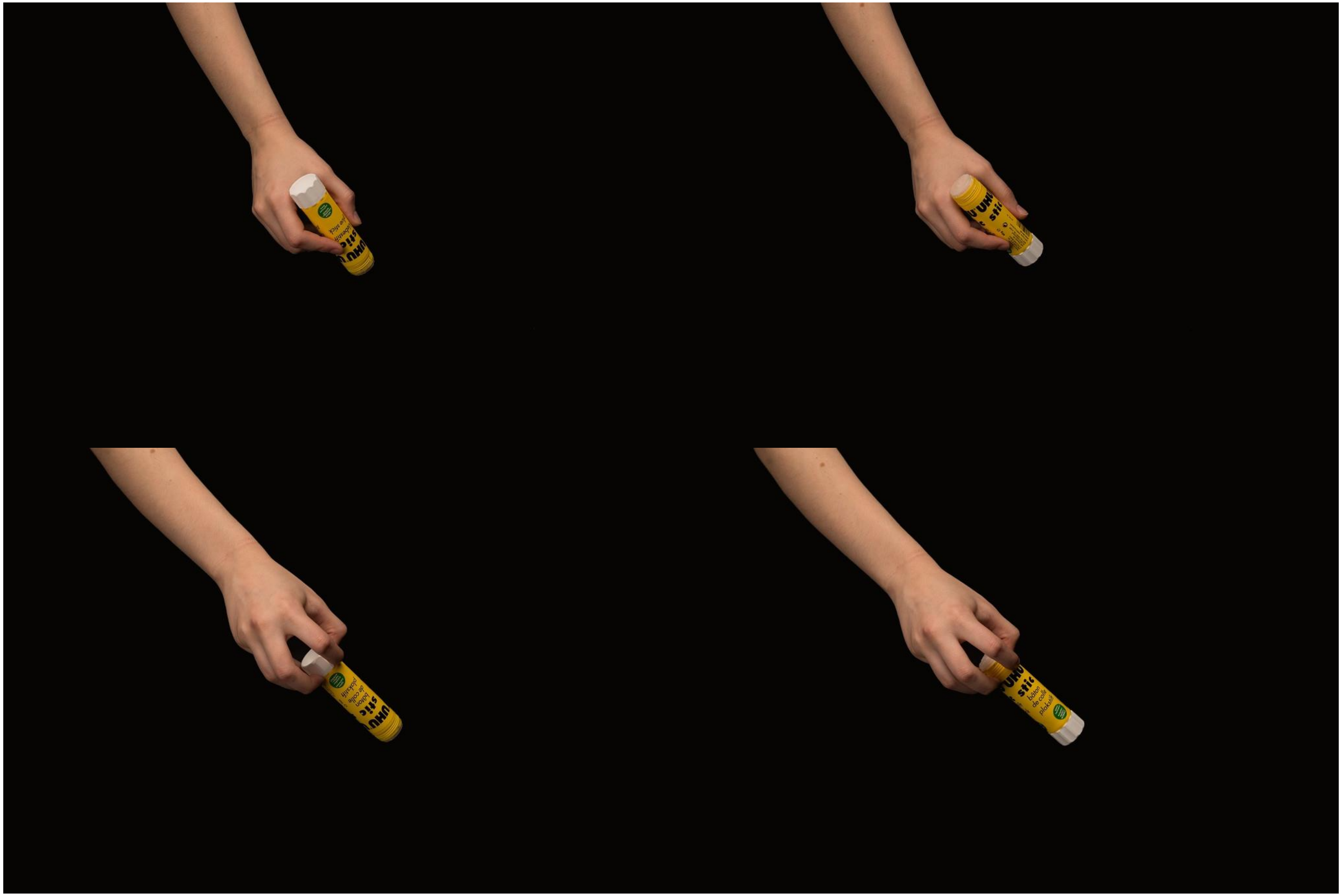

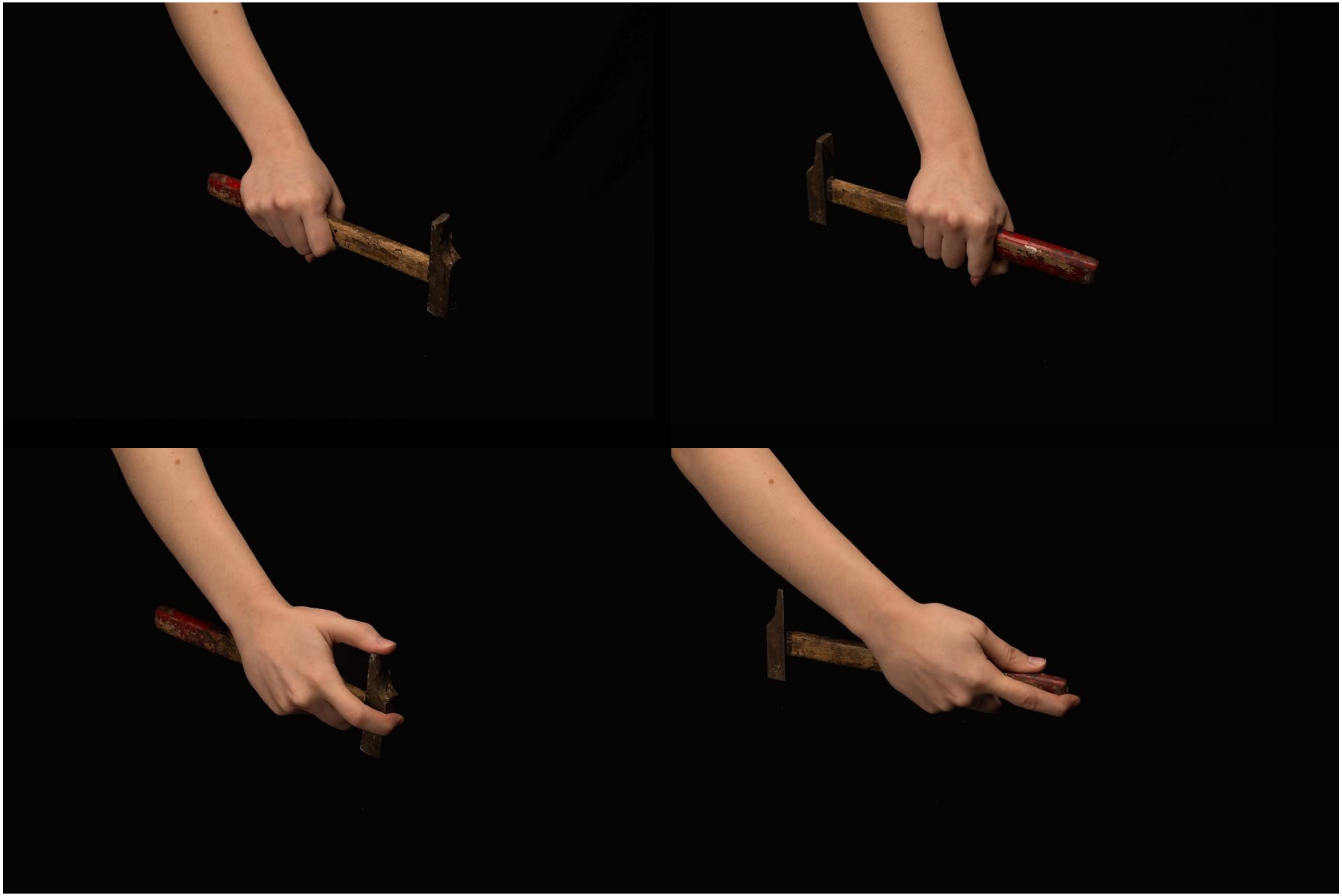

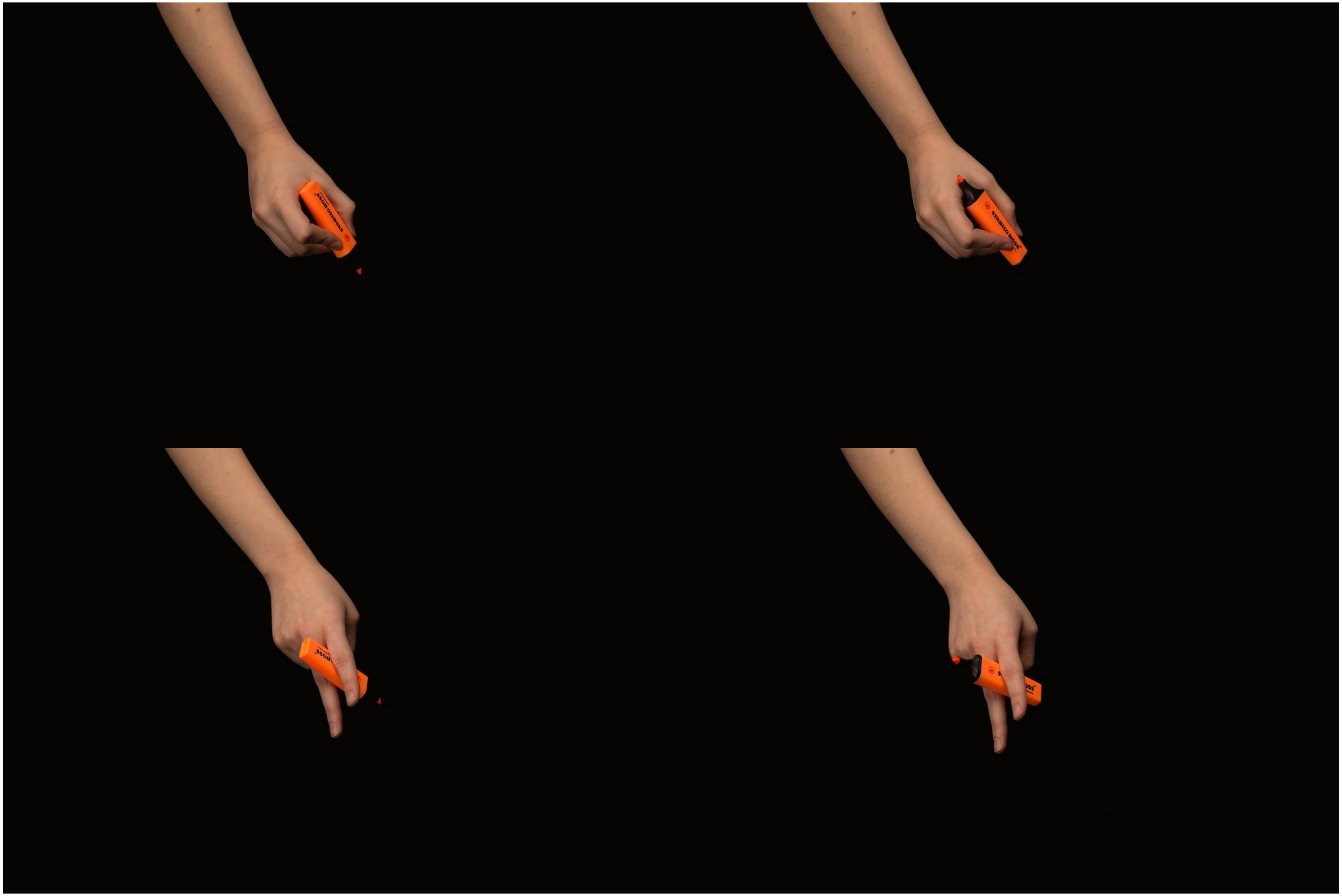

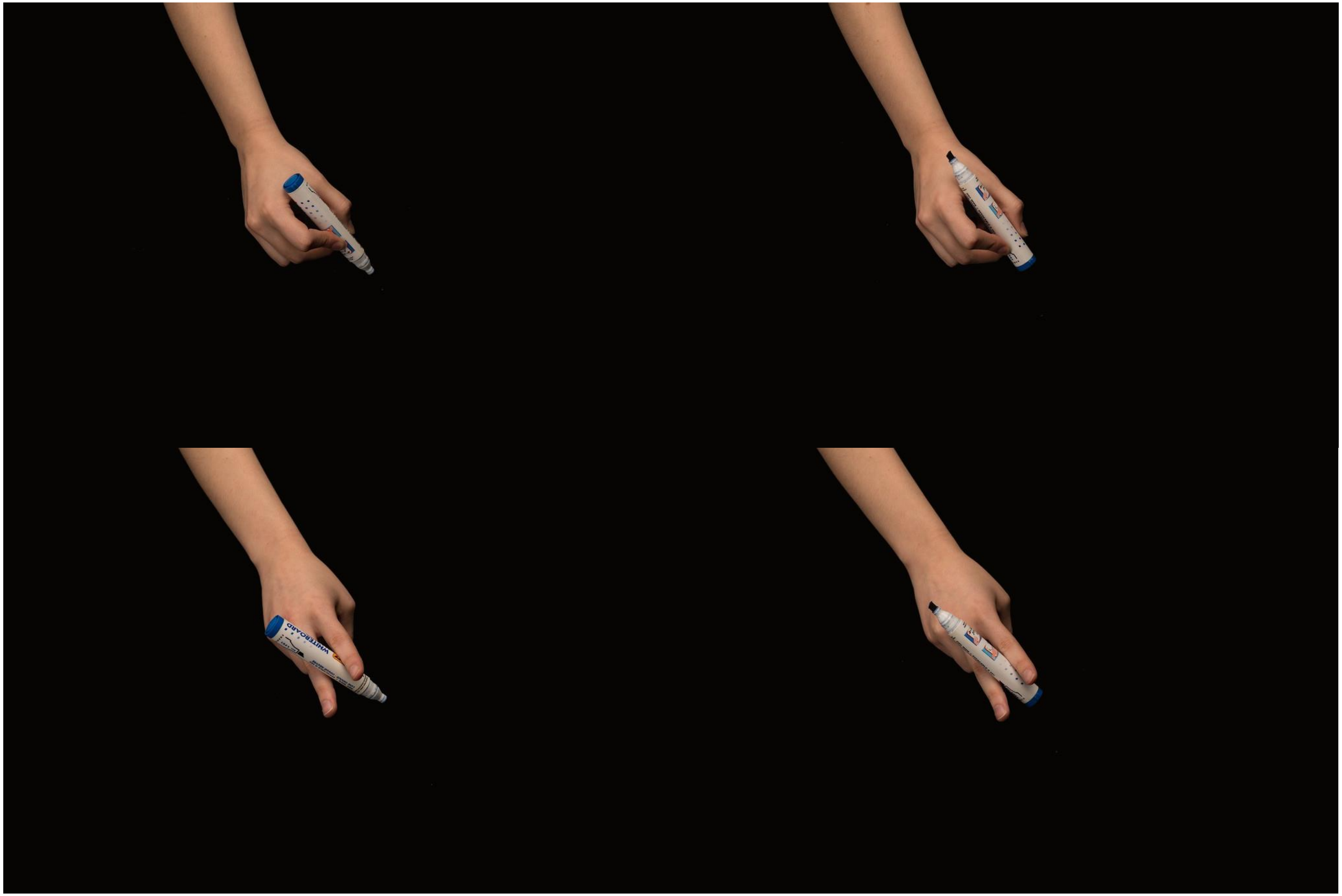
WHITEBOARD

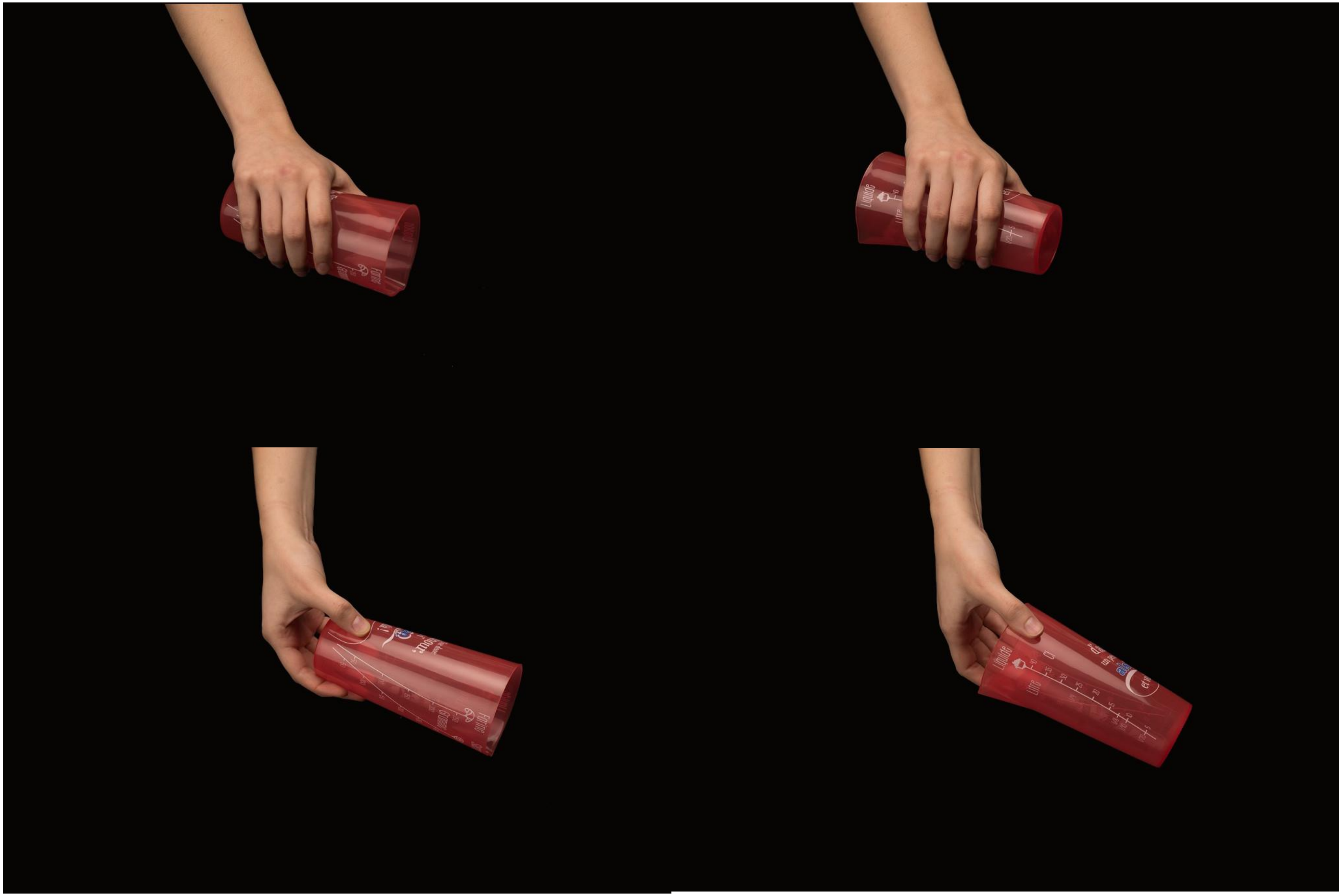

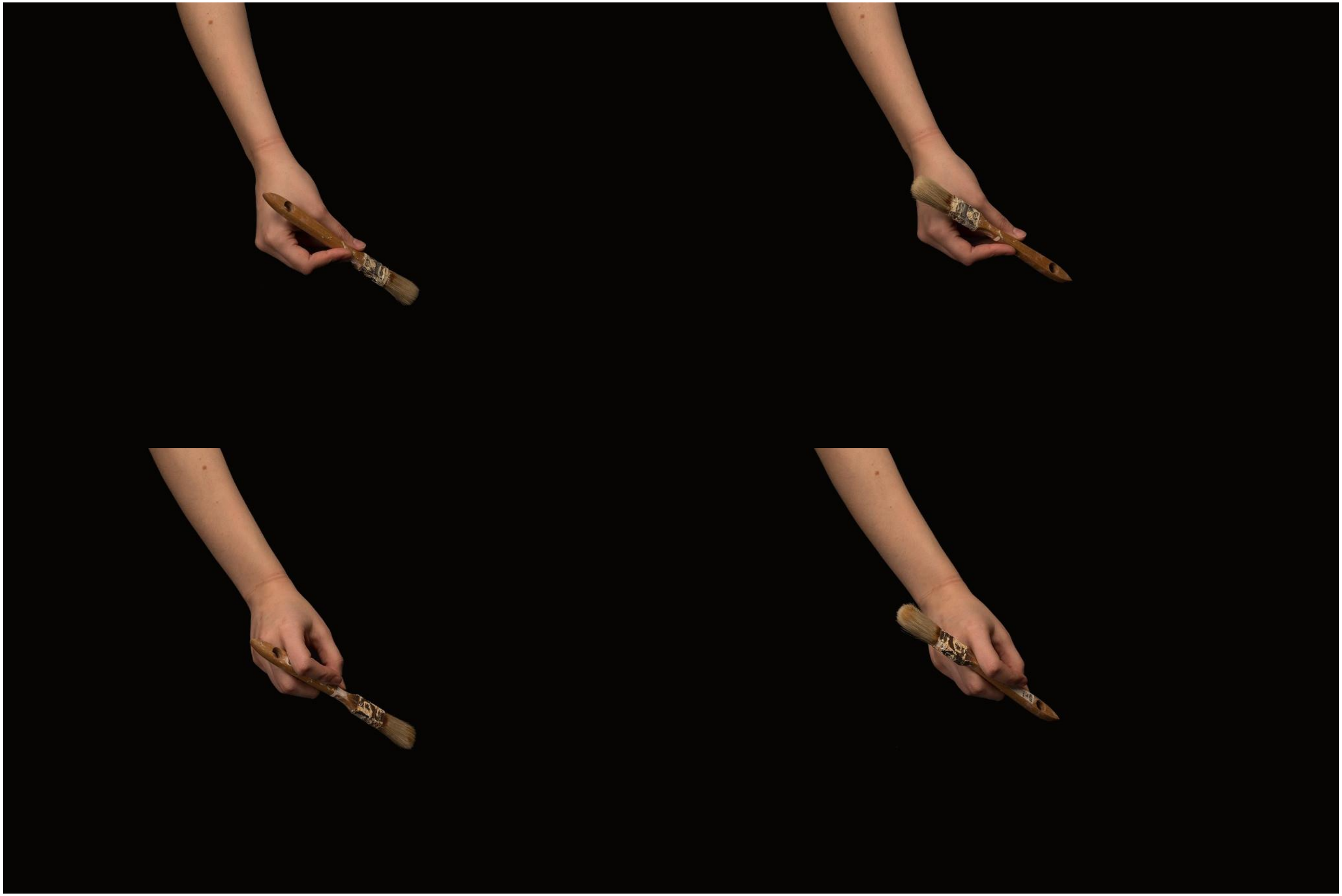

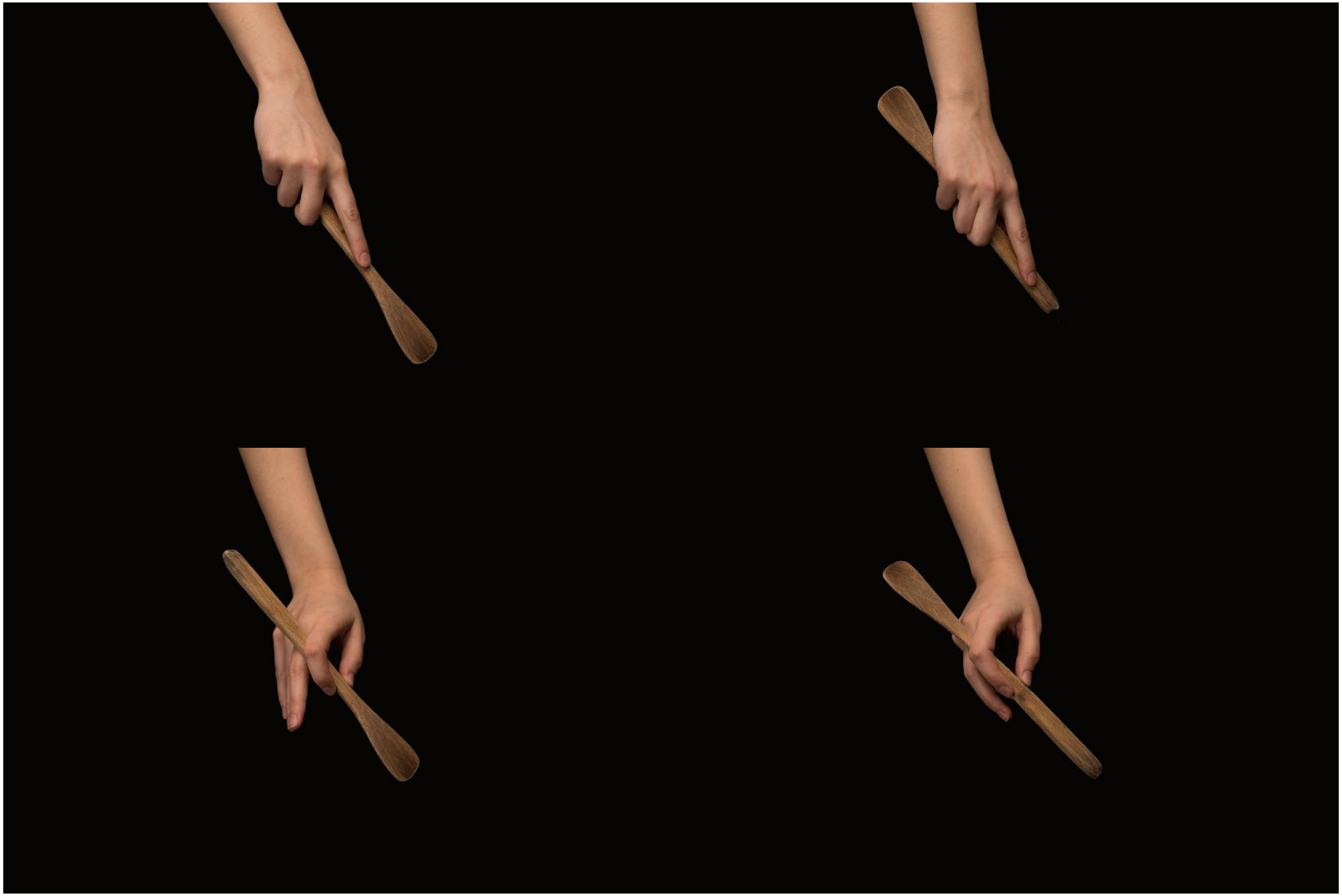

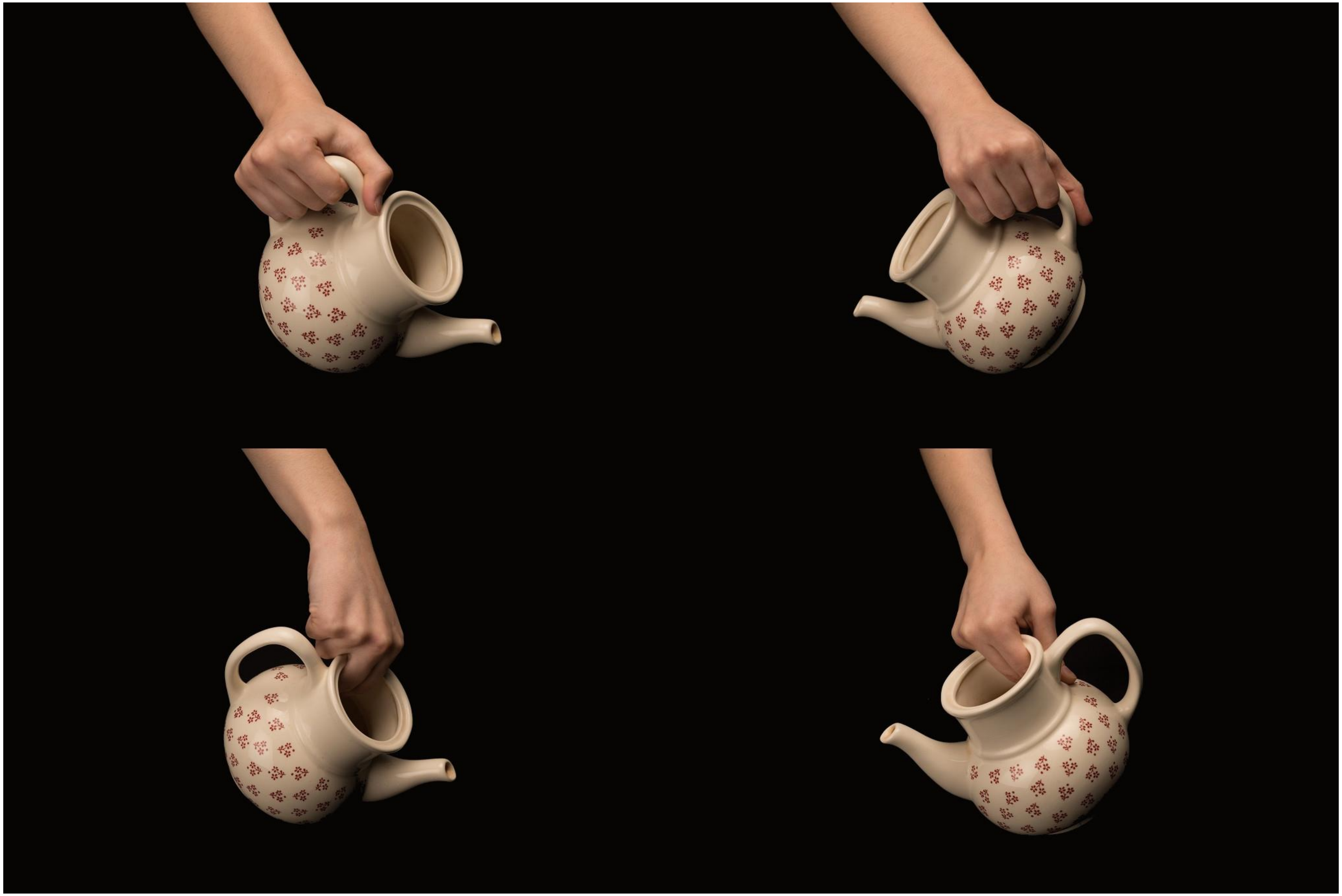

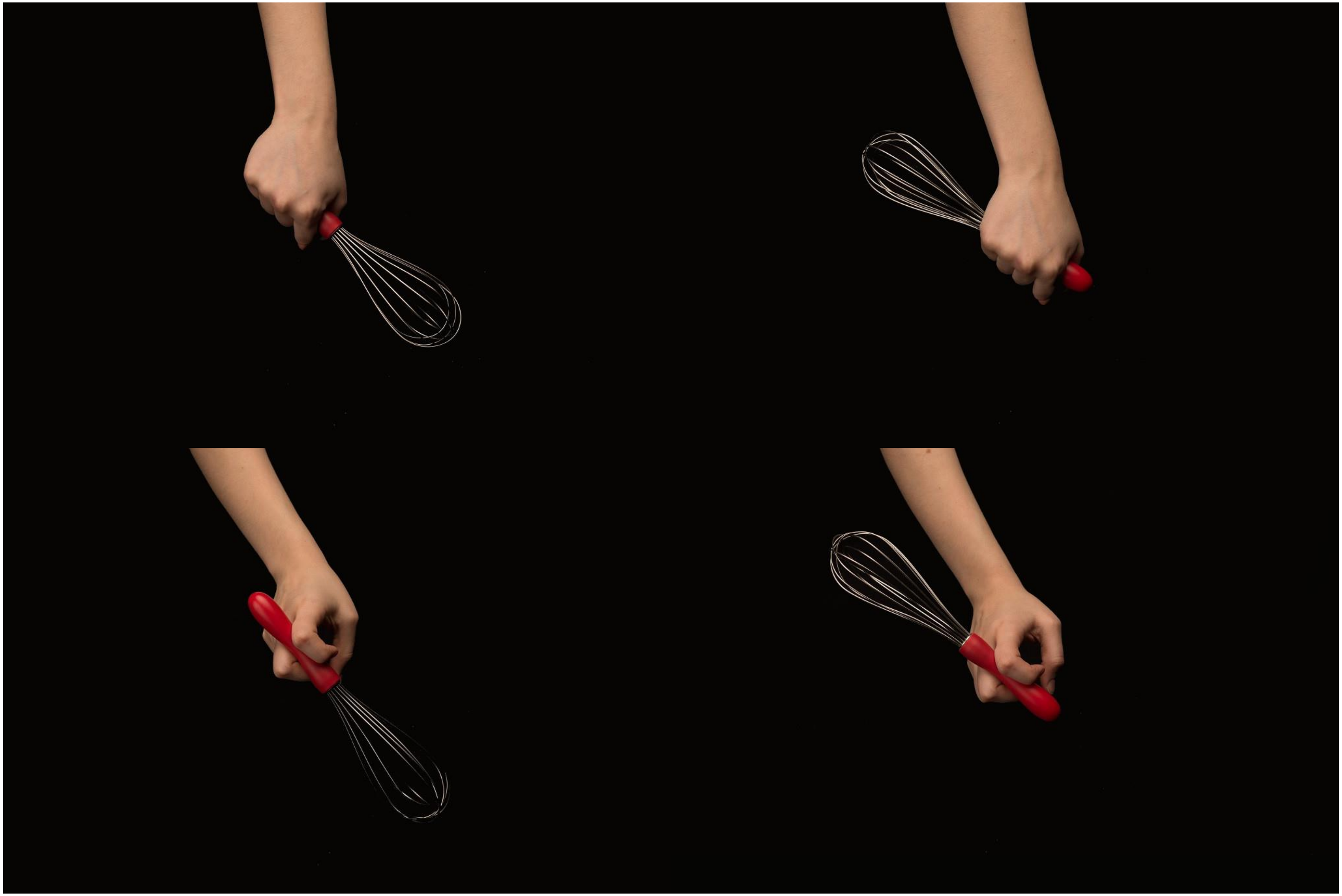

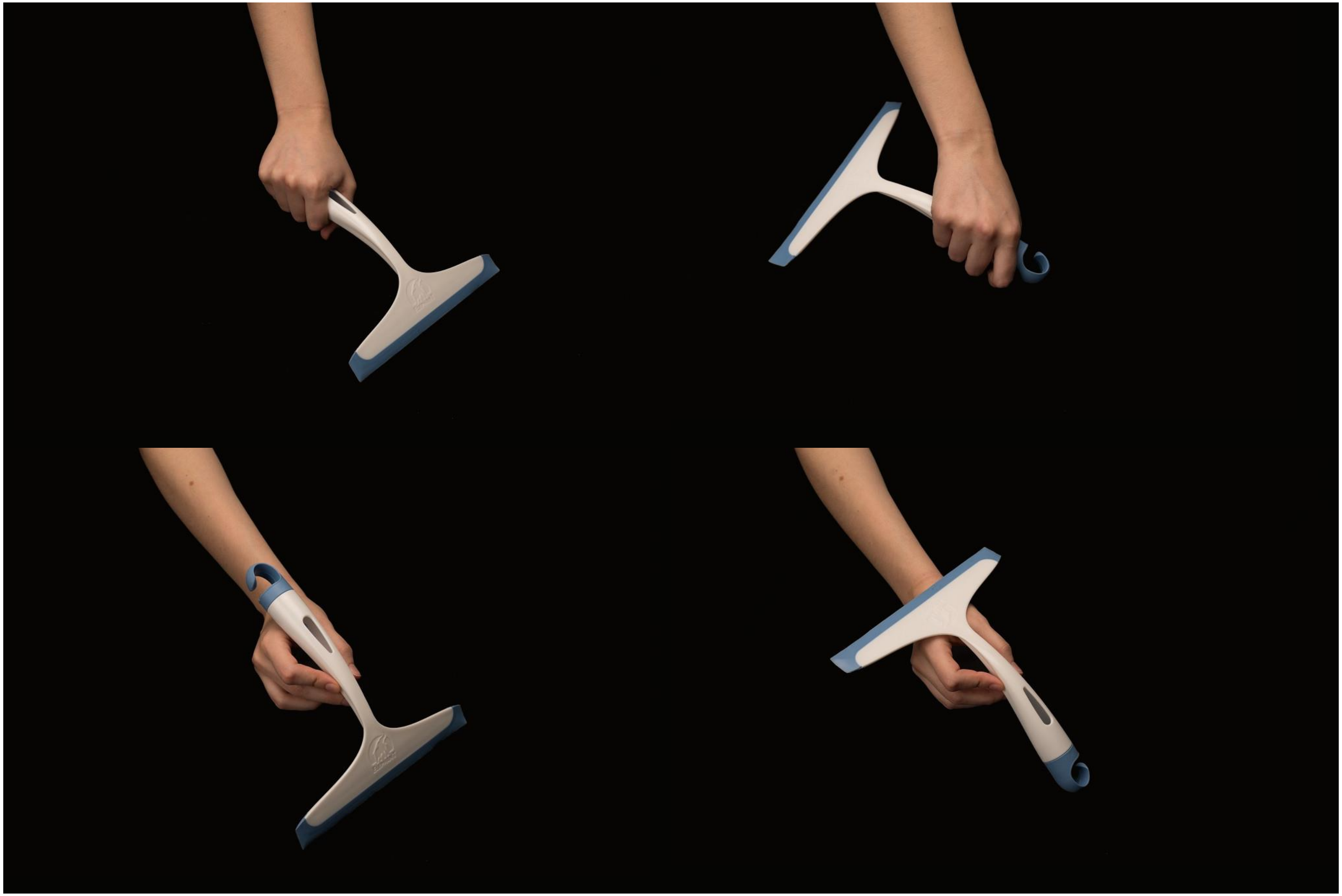

Supplement: Supplementary file 1 — Full set of stimuli. [file 41598_2020_61963_MOESM1_ESM.pdf]
